# MUC1-Driven Guanylin Gene Delivery via Succinylated PEI-9 Nanocarrier for Colorectal Cancer Treatment: An in Silico and In vitro Study

**DOI:** 10.34172/apb.025.45741

**Published:** 2025-10-15

**Authors:** Pouria Samadi, Fatemeh Rahbarizadeh, Fatemeh Nouri, Meysam Soleimani, Rezvan Najafi, Akram Jalali

**Affiliations:** ^1^Poursina Hakim Digestive Diseases Research Center, Isfahan University of Medical Sciences, Isfahan, Iran; ^2^Department of Medical Biotechnology, School of Advanced Medical Sciences and Technologies, Hamadan University of Medical Sciences, Pardis Campus, Shahid Fahmideh Street, P.O. Box, 6517838736, Hamadan, Iran; ^3^Department of Medical Biotechnology, Faculty of Medical Sciences, Tarbiat Modares University, Tehran, Iran; ^4^Department of Pharmaceutical Biotechnology, School of Pharmacy, Hamadan University of Medical Sciences, Hamadan, Iran; ^5^Research Center for Molecular Medicine, Hamadan University of Medical Sciences, Hamadan, Iran; ^6^Student Research Committee, Hamadan University of Medical Sciences, Hamadan, Iran

**Keywords:** Colorectal cancer, Guanylin, Guanylyl cyclase C, Gene therapy, Gene delivery

## Abstract

**Purpose:**

Addressing colorectal cancer (CRC) poses a significant challenge, demanding the precise delivery of therapeutic agents to eliminate cancer cells while minimizing the impact on healthy cells. The strategic selection of therapeutic targets, the utilization of nanocarriers with optimal efficacy and low toxicity, and the development of gene constructs with targeted expression in cancer cells are crucial aspects of this pursuit.

**Methods:**

This study employed a systems biology approach to comprehensively investigate the guanylin hormone-encoding gene (GUCA2A). Exploration encompassed expression patterns across tissues and single cells, clinical endpoints, methylation profiles, mutations, and immune and functional analyses. Subsequently, GUCA2A was identified as a potential target for gain of function studies, leading to its amplification and cloning into gene constructs featuring both a robust CMV promoter and a cancer-specific MUC1 promoter. The succinylated PEI-9, characterized by low toxicity and high gene transfer efficiency, was then fabricated and characterized on HCT-116 cancer cells and normal Vero cell lines.

**Results:**

systems biology studies revealed guanylin’s aberrant expression patterns, methylation variations, and mutational changes as well as its remarkable association with immune engagement and poor survival outcomes in CRC. Moreover, SPEI-9 was introduced as a highly efficient and safe nanocarrier for gene delivery purposes. Additionally, in vitro studies revealed that both guanylin-expressing gene constructs exhibited the potential to inhibit cell growth and proliferation, inducing apoptosis, suppressing cell migration, and curtailing colony formation. Notably, these effects were more robust but non-specific in cancer cells treated with constructs containing the CMV general promoter, while induction via the MUC1 promoter was more specific.

**Conclusion:**

A genetic construct featuring strong universal CMV and specific MUC1 promoter, expressing the guanylin peptide hormone, demonstrated highly effective and specific anticancer effects when transfected with nanocarriers characterized by high efficiency and low cytotoxicity. This nano-system holds promising implications for future targeted CRC therapy clinical trials.

## Introduction

 In recent decades, concerns over the side effects and limited efficacy of conventional treatments have driven the development of novel cancer therapies.^1^ With the rapid growth of multi-omics technologies and computational modeling over the past decade, in silico analyses have become essential for identifying novel therapeutic targets and potential drug candidates, particularly in cancer research.^2,3^

**Figure 1:**
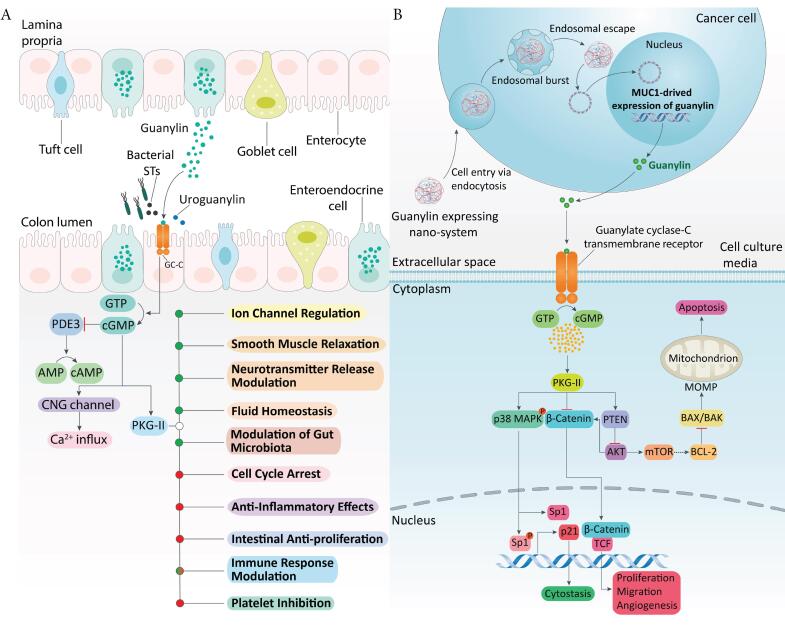
GC-C signaling in the intestine and engineered guanylin nano-system. (A) Under physiological conditions, guanylin and uroguanylin, or bacterial heat-stable toxins, activate GC-C on intestinal epithelial cells, leading to cGMP production and PKGII activation. This pathway regulates ion transport, fluid balance, barrier integrity, cell proliferation/differentiation, and exerts anti-inflammatory and immunomodulatory effects, with implications in CRC and microbiota modulation. Positive and negative regulations are indicated in green and red. (B) Schematic of the guanylin-expressing nano-system. Following endocytosis and endosomal escape, the construct drives guanylin expression, secretion, and GC-C activation. This elevates cGMP, activating PKGII and p38 MAPK, which phosphorylate Sp1 to upregulate p21 and induce cytostasis, while counteracting β-catenin/TCF and PTEN/Akt pro-survival signaling

 The most prevalent occurrence in colorectal cancer (CRC), ~70–80%, involves the inactivation of the tumor suppressor gene adenomatous polyposis coli (*APC*). This co-occurs with the activation of oncogenic *KRAS* (40–50%), and the presence of mutations in other tumor suppressor genes, such as *PTEN* or *TP53*, or oncogenes like *PIK3CA*, is also frequently observed.^4^ In our earlier study,^5^ we conducted a rigorous integrative transcriptome analysis across multiple CRC datasets and identified *GUCA2A*, encoding the endogenous peptide hormone guanylin (*GUCA2A)*, as the most consistently and significantly downregulated gene among a final set of 37 differentially expressed genes (DEGs). Notably, *GUCA2A* also exhibited the highest degree of connectivity in our regulatory interaction network, highlighting its central role in CRC-associated transcriptomic alterations. Previous research has shown that *GUCA2A* experiences loss after *APC* inactivation in mouse models featuring conditional biallelic Apc deletion (Apc^CKO/CKO^) and Apc loss of heterozygosity (Apc^min/+^).^6^ Given that guanylin is a peptide stimulant for *GUCY2C*, encoding a member of the family of transmembrane receptor guanylyl cyclases, it fosters cyclic guanosine monophosphate (cGMP) accumulation, which in turn, facilitates electrolyte and fluid secretion within the large intestine as well as many other vital roles summarized in Figure 1A.^7,8^ Beyond this, *GUCY2C* regulates essential homeostatic processes that are often dysregulated during tumorigenesis, including cellular functions like metabolism, proliferation, and differentiation programs.^9,10^ Silencing *GUCY2C* is a universal characteristic of colorectal tumorigenesis, which contributes to the promotion of crypt hyperplasia, acceleration of the cell cycle, induction of DNA damage, and higher susceptibility to tumor development.^11,12^ Most tumor subtypes maintain the presence of cell-surface *GUCY2C* expression as they progress through different stages of the disease.^13,14^ However, transformation universally orphans the receptor due to the depletion of endogenous hormones. These observations suggest reactivating endogenous hormone generation via gene therapy approaches, which may be a novel therapeutic strategy for CRC.^15^

 Beyond APC-driven transcriptional repression, guanylin expression is also regulated by key pathways implicated in CRC progression, including Wnt/β-catenin, Notch, and inflammatory signaling cascades. Dysregulation of these networks further contributes to the silencing of GUCA2A and impairment of GUCY2C signaling during neoplastic transformation. Restoring guanylin signaling may not only counteract the loss of epithelial homeostasis but also synergize with therapies targeting complementary oncogenic pathways. Collectively, these observations support gene therapy strategies to re-establish guanylin expression, re-activate GUCY2C signaling, and restore epithelial balance in CRC.^16^

 Gene therapy seeks to treat genetic diseases by introducing therapeutic genetic material into cells to modify their function.^17^ A central challenge is achieving precise and efficient delivery, which relies on specialized carriers known as vectors. These vectors are broadly classified as viral or non-viral. Viral vectors—such as adenoviruses, lentiviruses, and adeno-associated viruses (AAV)—provide high transduction efficiency but carry risks of immunogenicity and insertional mutagenesis. Non-viral systems, including lipid nanoparticles and polymer-based carriers, offer safer profiles and simpler production but typically show lower delivery efficiency.^17^

 A promising way to advance cancer gene therapy is through precision-targeted expression using tissue- or tumor-specific promoters.^18^ Conventional promoters such as hTERT, Survivin, uPAR, COX-2, and more recently MUC1, are expressed at significantly higher levels in various cancers—particularly gastrointestinal (GI) cancers—compared with normal tissues.^19^ The MUC1 promoter, expressed in over 90% of CRC cells, offers a highly specific and potent alternative to conventional promoters for CRC gene therapy. Furthermore, placing a hypoxia-responsive element (HRE) upstream of the MUC1 promoter enhances tumor-specific gene expression, particularly under the hypoxic conditions typical of cancer cells.^20,21^

 Polyethylenimine (PEI) and related polycations are widely studied non-viral gene carriers due to their ability to form stable electrostatic complexes with nucleic acids. These polyplexes protect plasmid DNA from enzymatic degradation and promote more efficient intracellular delivery.^22^ While PEI has demonstrated efficacy as a gene carrier, its application at higher doses has raised concerns due to its inherent toxicity. Although PEI has proven effective as a gene carrier, its use at higher doses raises concerns due to intrinsic toxicity. This toxicity arises from PEI’s strong positive charge, which promotes intense interactions with cell membranes and can lead to cellular damage. To overcome this issue, research has increasingly focused on modifying the polymeric backbone of PEI to reduce its positive charge, thereby decreasing cytotoxicity. Such modifications offer the potential to improve the safety profile of PEI, maintaining its effectiveness as a gene carrier while minimizing harmful effects at higher concentrations.^21^

 Succinylated PEI refers to PEI that has been chemically modified by attaching succinyl groups. This modification involves linking succinyl moieties—a type of dicarboxylic acid—to the polymer backbone of PEI. One key outcome of succinylation is the reduction of PEI’s overall positive charge. Unmodified PEI carries a strong positive charge, which can interact with cell membranes and cause cytotoxic effects. By introducing succinyl groups, the charge density is lowered, resulting in a less positively charged, and therefore less cytotoxic, polymer. Succinylated PEIs have garnered interest for gene delivery applications. This chemical modification aims to retain PEI’s inherent ability to form complexes with nucleic acids while mitigating its cytotoxicity. As a result, succinylated PEIs often show improved biocompatibility, reduced toxicity, and enhanced efficiency in delivering genetic material to target cells, making them promising candidates for gene therapy.^23^ SPEI-9, a succinylated derivative of PEI with a low degree of succinylation (~9% by polymer weight), exhibits reduced charge density, significantly lower cytotoxicity, and enhanced gene transfer efficiency compared to unmodified PEI, making it an optimal and promising vector for gene therapy applications.^24,25^

 In this study, we first analyzed transcriptomic data from CRC, pan-cancer datasets, and a range of human tissues and single-cell types to characterize GUCA2A expression patterns. Based on these findings, we developed a gene therapy approach aimed at restoring endogenous guanylin hormone expression, employing MUC1 as a tumor-specific promoter and CMV as a universal promoter for targeted CRC therapy. To facilitate efficient gene delivery, we synthesized and characterized SPEI-9, a novel nanocarrier. We then assessed the downstream anti-tumor effects of this guanylin-expressing nanosystem in both CRC and normal cell lines. A schematic overview of the proposed therapeutic mechanism is presented in Figure 1B.

## Materials and Methods

###  Exploration of GUCA2A gene expression patterns

 We leveraged the Tabula Muris (https://tabula-muris.ds.czbiohub.org) and Single Cell Portal (https://singlecell.broadinstitute.org) databases to explore the spectrum of GUCA2A and MUC1 gene expression across various human tissues, as well as within distinct epithelial cell populations in CRC and their adjacent normal counterparts. Furthermore, we performed differential expression analyses to examine changes in GUCA2A expression across both microarray and The Cancer Genome Atlas (TCGA) datasets. Expression data were obtained from the comprehensive Cancer Cell Line Encyclopedia (CCLE) project (GSE36133, n = 55 CRC cell lines) and a clinically homogeneous TCGA COAD-READ cohort (normal = 51, CRC = 644). All analyses were conducted using the LIMMA and edgeR packages in R.^26,27^

###  Assessing GUCA2A expression as a prognostic indicator in CRC

 To assess the prognostic significance of *GUCA2A* expression on patient outcomes, we conducted univariate Cox regression analyses. Our investigation involved different CRC datasets from multiple microarray studies and TCGA. This comprehensive approach allowed us to make predictions concerning different clinical endpoints, including cancer-specific survival (CSS), disease-free interval (DFI), disease-free survival (DFS), disease-free metastasis survival (DFMS), disease-specific survival (DSS), overall survival (OS), progression-free interval (PFI), progression-free survival (PFS), and relapse-free survival (RFS) across diverse CRC datasets. Additionally, we analyzed RNA-seq and clinical data from the TCGA COAD and READ cohorts, obtained via the UCSC Xena platform. Expression data were pre-processed as transcripts per million (TPM) and log-transformed as log2(TPM + 1) for normalization. Only primary tumor samples with complete OS information were included (n = 611). Patients were divided into high and low *GUCA2A* expression groups using the maxstat algorithm (from the maxstat R package), identifying the optimal expression cutoff that maximizes the log-rank statistic for OS separation. Kaplan-Meier survival curves were generated, and differences in survival assessed using the two-sided log-rank test. A *P* value < 0.05 was considered statistically significant. These analyses were performed using the R packages survival, survminer, maxstat and ggplot2.

###  Mutation and methylation profile analysis

 To comprehensively investigate the mutational landscape of *GUCA2A* across various cancer types, we utilized the capabilities of the cBioPortal tool (http://www.cbioportal.org/) and the BEST tool. Focusing our efforts on the “TCGA Pan-Cancer Atlas Studies” cohort, we conducted an extensive investigation. This analysis encompassed the assessing of specific mutation sites, genetic alteration frequencies, and mutation types influencing *GUCA2A*. Furthermore, we utilized methylation data from the SMART App (http://www.bioinfo-zs.com/smartapp) to investigate the relationship between *GUCA2A* expression and methylation patterns within the TCGA COAD-READ dataset. Box plot visualizations generated using the ggplot2 package in R.

###  Relationship between GUCA2A expression and immunity

 We also investigate the potential link between the association of *GUCA2A* expression and the tumor microenvironment (TME) in pan-cancer. To achieve this, we assessed various parameters, including stromal score, ESTIMATE score, immune score, tumor purity, and immune-related pathways. Multiple algorithms, such as XCELL, QUANTISEQ, CIBERSORT-ABS, EPIC, and TIMER, based on the data retrieved from the BEST tool (https://rookieutopia.com/app_direct/BEST/).^28^ To visualize the results, we utilized the ggplot2 R package. The generated heat maps provided insights into the relationships between *GUCA2A* expression, the above metrics, and immune infiltrating cells across different cancers.

###  GSVA and correlation functional analyses

 Gene set variation analysis (GSVA) was performed using the R package “GSVA” (v1.44.5) on log₂(TPM + 1) normalized expression data from TCGA-COADREAD. We used the 50 hallmark gene sets from MSigDB v7.5.1 to calculate pathway enrichment scores for each CRC sample. Samples ordered by *GUCA2A* expression, and Pearson correlation coefficients computed between *GUCA2A* levels and pathway scores. Pathways with |r| > 0.3 and *P*< 0.01 considered significantly associated with *GUCA2A* expression. Moreover, a list of top correlated DEGs was subjected to functional enrichment analysis using the clusterProfiler R package. Gene Ontology (GO) to determine the biological processes, molecular functions, cellular components, and pathways significantly associated with the DEGs. Enrichment was deemed significant at an adjusted *P* value < 0.05. Also, a heatmap was generated using the ggplot2 R package to visualize the expression patterns of the top correlated DEGs (low and high expression groups) across samples.

###  Assessing drug sensitivity of GUCA2A

 To investigate the correlation between *GUCA2A* expression and drug sensitivity across cancer types, we utilized publicly accessible bioinformatics tools, CPADS (Cancer Pan-drug Sensitivity Analysis, https://smuonco.shinyapps.io/CPADS/) and the BEST web tool for cancer-specific drug response data. All data were processed, analyzed, and visualized using R software, which included correlation analyses and the generation of heatmaps to display relationships between *GUCA2A* expression and drug response.

###  ROC curve analysis of GUCA2A

 Expression data for *GUCA2A* and clinical metadata, including MSI and TMB status, were obtained from TCGA-COAD-READ. The diagnostic performance of *GUCA2A* expression for distinguishing CRC samples from normal tissue was assessed using ROC curve analysis using the log2(TPM + 1) expression data from TCGA-COAD dataset. Samples were categorized into two groups: CRC tissues (cases) and normal tissues (controls). The area under the ROC curve (AUC) was calculated to measure discriminatory performance, and 95% confidence intervals (CIs) were estimated using DeLong’s method. The Youden index was used to determine the optimal threshold for sensitivity and specificity. ROC curves were generated using the pROC package in R, which computes the area under the curve (AUC) as a metric of diagnostic ability.

###  GUCA2A correlation between TMB and MSI

 The association between *GUCA2A* expression and microsatellite instability (MSI) status was assessed using Wilcoxon rank-sum tests to compare expression levels between MSI-H and MSS groups. For tumor mutational burden (TMB), Spearman correlation coefficients calculated to determine the strength and significance of the relationship between *GUCA2A* expression and TMB as a continuous variable. The ggstatsplot package in R was used for visualizations.

###  Amplification of GUCA2A coding sequence

 The primer for the amplification of the *GUCA2A* coding sequence (CDS) was designed using the primer3plus online tool, then it was analyzed with the primer blast, Multiple Primer Analyzer, and IDT OligoAnalyzer Tool to check the optimality of various parameters. The Kozak sequence was placed at the beginning of the Forward (F) primer to initiate translation. Also, the cut site of *BamHI* enzyme was placed at the 5’ end of the primer before the Kozak sequence and the *XbaI* enzyme cut site was placed at the 5’ end of the Reverse (R) primer (Table 1). Following the successful PCR amplification of the *GUCA2A* CDS from CRC normal tissue-derived cDNA, the resulting fragment was subjected to purification using the AccuPrep® PCR/Gel Purification Kit (Bioneer, Korea) following the manufacturer’s protocol. Subsequently, enzymatic digestion utilizing *BamHI* and *XbaI* restriction enzymes was employed to process the digested fragment, facilitating the removal of undesired cleavage sites through additional gel extraction steps.

**Table 1:** Characteristics of primers and oligomers used in amplification and RT-qPCR

**Product size**	**Gene ID**	**Antisense strand**	**Sense strand**	**Gene**
369 bp	2980	TTCTCTAGACTAGCATCCGGTACAGGCAG	ACGGGATCCGCCATGAATGCCTTCCTGCTCTCC	*GUCA2A* CDS for cloning
117 bp	2980	GGTTGCTACAGAGGATGG	TGGAGTCAGTGAAGAAGC	*GUCA2A*
248 bp	2597	GCGTCAAAGGTGGAGGAGTGG	AAGGCTGTGGGCAAGGTCATC	*GAPDH*
188 bp	7431	CGTTGATAACCTGTCCATC	CATTGAGATTGCCACCTAC	*VIM*
100 bp	1000	CCCACAATCCTGTCCACATC	ATTCGGGTAATCCTCCCAAATC	*CDH2*
138 bp	1026	AGTCGAAGTTCCATCCCTCA	ATGTCCGTCAGAACCCATGC	*CDKN1A*
98 bp	1499	CCTTCCATCCCTTCCTGTTTAG	CTTCACCTGACAGATCCAAGTC	*CTNNB1*
198 bp	581	TGTCCAGCCCATGATGGTTC	CAGAGGCGGGGGATGATTG	*BAX*
121 bp	596	GTCTACTTCCTCTGTGATGTTGTAT	TGGAGAGTGCTGAAGATTGA	*BCL2*

###  Construction of the guanylin-expressing vectors 

 In this investigation, the mammalian expression vector, pCDNA 3.1/Hygro( + ) (Invitrogen), was selected as the basic genetic construct. The MUC1 promoter and a cassette containing the hypoxia response element (HRE) were amplified and inserted into the pcDNA3.1/Hygro ( + ) basic vector to replace the CMV promoter, resulting in the HRE-pMUC1-Insert construct. This construct was kindly provided by Dr. Rahbarizadeh’s laboratory. The HRE-p*MUC1*-mRNA, alongside the default pCMV-mRNA vector, were both prepared with *BamHI*/*XbaI *flanking cutting sites for subsequent subcloning procedures. To propagate these vectors, GM2163 bacteria (Dam^-^ Dcm^-^), a derivative of E. coli strain K12, were employed, as the *XbaI *cut site is hindered by dam methylation. Following bacterial transformation, the vectors were extracted using the GeneJET Plasmid Miniprep kit. Following enzymatic digestion with *BamHI* and XbaI, a further gel extraction step was performed to prepare vectors for downstream procedures. The digested vectors and *GUCA2A* CDS fragment were subsequently ligated together. After successful transformation, colony selection was carried out via colony PCR, followed by validation through Sanger sequencing. The resulting vectors, named HRE-p*MUC1*-*GUCA2A* and pCMV-*GUCA2A*, were then prepared for subsequent cell culture analyses.

###  Synthesis and structural characterization of SPEI-9

 PEI (0.5 g) was dissolved in 8.5 mL of water and 1.5 mL of a NaCl solution (3 M). The pH of the solution was then adjusted to 5 using 1 M HCl. Precise quantities of succinic anhydride (0.1 M, for 9% modification) were dissolved in dimethyl sulfoxide (DMSO) and carefully added dropwise to the PEI solution. The reaction was conducted at room temperature for a duration of 3 h. To purify the crude products, a dialysis was performed using a 10,000-12,000 molecular weight cutoff membrane. Initially, dialysis was carried out against a 0.25 M NaCl solution to eliminate unreacted succinate. Subsequently, the solution was dialyzed twice against water at 4 °C to remove residual salt. Following the dialysis process, the aqueous solution was subjected to lyophilization. A schematic diagram of the reaction of succinic anhydride and basic PEI to make SPEI-9 is displayed in Supplementary file 1, Figure S1. For the downstream tests (except structural analysis), we prepared the polymers in different concentrations with HBG buffer (20 mM HEPES in 5% glucose solution, pH 7.2) to obtain different C/P ratios.

 The degree of modification was assessed using 1H nuclear magnetic resonance (NMR) spectroscopy (Varian INOVA 500MHz, Palo Alto, USA) in deuterium oxide (D2O). The presence of carboxylic acid changes on the surface of SPEI-9 was also confirmed using Fourier Transform Infrared (FT-IR) (Agilent-USA-Cary 680). The spectra were analyzed using Origin software (version 9.85).

###  The buffering capacity of PEI and SPEI-9 nanocarriers 

 PEI-based nanocarriers exhibit robust pH resistance within a broad range (pH 2 to 10). This resistance eventually leads to an increase in the osmotic pressure, its bursting, and the release of the polyplex into the cytosol due to the proton sponge effect. To assess the buffering capacity of both PEI and SPEI-9, a 2 mg/mL solution of the nanocarriers was initially dissolved in deionized water, and its pH was measured. The solution’s pH was initially adjusted to 12 using 1N NaOH, followed by gradual titration with 5 μL increments of 1N HCl until the pH dropped below 2.5. A pH titration curve was then constructed based on the volume of acid added. Deionized water served as the negative control, while PEI was used as the positive control.

###  Preparation and loading efficiency of PEI and SPEI-9 polyplexes 

 PEI/DNA and SPEI-9/DNA polyplexes were prepared by adding 50 μL of the polymer solution in different concentrations to 50 μL of the gene construct with the same concentration (at concentration of 40 μg/mL in HBG buffer). After gently pipetting the mixture (10-20 times), it was allowed to incubate for 20-30 min at room temperature to form stable complexes. To assess the binding affinity of PEI and SPEI-9 polymers with genetic constructs, a gel retardation assay was employed. Polyplexes were prepared at various C/P ratios ranging from 0.25 to 8. Gel electrophoresis was subsequently conducted, and the results were analyzed using a Gel-Doc device.

###  DNase degradation assay

 To assess the protective ability of PEI and SPEI-9 against the enzymatic degradation of loaded DNA by serum nucleases, a DNase protection test was conducted. Polyplexes were prepared at various C/P ratios (ranging from 0.25 to 8) and exposed to 1 μL of DNase I enzyme (1 U/ μL) in PBS or DNase/Mg2 + reaction buffer for 30 min at 37 °C. Then, 4 μL of 50 mM EDTA was added to deactivate the enzyme by removing the Mg2 + ions in the enzyme buffer. All microtubes were then incubated for 10 min at 65°C to inactivate the enzyme. Subsequently, 10 μL of 1 mg/mL heparin was added to facilitate the separation of the DNA from the nanocarrier. The microtubes were further incubated for 2 h at room temperature. Finally, the samples were subjected to electrophoresis in a 1% agarose gel.

###  Measurements of the size and zeta potential of the polyplexes

 The average hydrodynamic particle size and surface charge density of polyplexes were measured by Dynamic Light Scattering and Laser Doppler Velocimetry by Malvern Nano Zetasizer (Malvern, UK) and results were reported as mean ± SEM.

###  Hemolysis test of polyplexes

 The hemolysis assay was conducted using human blood to assess the blood compatibility of the synthesized polymers. Arterial blood was collected, and red blood cells (RBCs) were isolated through centrifugation (3000 rpm for 10 min) and washing with PBS. Washed RBCs were then exposed to polyplexes (using 100 μL of washed RBCs) with various C/P ratios, with deionized water and PBS serving as positive and negative controls, respectively. The samples were incubated at 37 °C for 2 h, followed by centrifugation (13,000 rpm for 10 min), and the absorbance of the supernatant (A) was measured at 540 nm. The percentage of hemolysis was calculated as follows.


$$Hemolysis\;(\%)=\frac{A\;sample-\;A\;negative}{A\;positive-\;A\;negative}\times 100$$

###  Protein Interaction

 To evaluate nonspecific protein binding interactions, 0.5 mL of bovine serum albumin (BSA) standard solution (2 mg/mL) was mixed with 0.5 mL of each polyplex solution (using 1 mg/mL of polymers). These mixtures were incubated at 37 °C for 1 h, followed by centrifugation to collect supernatant samples. The protein concentrations in these samples were quantified using a BCA assay with a BSA calibration curve. The parameter A, representing protein interaction, was defined as:


$$A=1-\frac{CsVs}{CiVi}$$

Here, *Ci* represents the initial BSA concentration (2 mg /mL), *Cs* is the BSA concentration in the supernatant determined by the BCA assay, Vi is the initial volume of the BSA solution (0.5 mL), and Vs is the total volume of the BSA solution after the adsorption measurement (1 mL). Interaction value A quantifies the extent to which protein has been removed from the initial solution through interaction with the polymer. It ranges from 0 (indicating no removal of protein) to 1 (representing complete protein removal).

###  Evaluation of the gene-hormone therapy nano-system in vitro 

 After designing, constructing, and validating the therapeutic vectors, pCMV-*GUCA2A* and HRE-ERE-pMUC-*GUCA2A*, as well as SPEI-9 (C/P 4) as a potent gene delivery nano-system, we evaluate the therapeutic nano-system through different *in vitro* assays on two cell lines of HCT-116 (National Cell Bank, Pasteur Institute of Iran, Tehran, Iran) as CRC cell line and the Vero (National Cell Bank, Pasteur Institute of Iran, Tehran, Iran), as a normal African green monkey kidney cell line. Cells were grown in Dulbecco’s modified eagle’s medium (DMEM) medium (Bioidea, Iran) supplemented with 10% fetal bovine serum (FBS, Gibco, USA), 1% Penicillin/Streptomycin (Gibco, USA), and were maintained in an incubator at 37 °C and 5% CO_2_.

###  Cell culture and transfection of the nano-system

 On the first day, 1 × 10^5^ HCT-116 and Vero cells were seeded in separate wells of 12-well plates. The following day, for cell transfection, a mixture of 50 μL PEI and SPEI-9 nanocarriers with a pEGFP-N1 gene construct (40 ng/μL stock) encoding enhanced green fluorescent protein (EGFP) was created. The mixture was combined with 50 μL of FBS-free medium, vortexed for 10 s, mixed by pipetting, and incubated for 30 min to allow polyplex formation. Afterward, 100 μL of complete medium was added dropwise to each well, and the plates were transferred to the incubator. Six to eight hours post-transfection, the medium was replaced with 1 mL of complete culture medium containing FBS. Cells were then incubated for 24, 48, and 72 hours. Transfection efficiency was assessed using a fluorescence microscope to determine the optimal time point for subsequent experiments.

###  Assessment of mRNA expression levels for guanylin and downstream genes 

 Following transfection of 3 × 10^5^ HCT-116 and Vero cells with different groups (pCMV-*GUCA2A*, HRE-p*MUC1*-*GUCA2A*, pEGFP-N1, and control) using SPEI-9 in a 6-well plate, we examined alterations in the expression of the *GUCA2A* gene and its downstream targets, specifically β-catenin (*CTNNB1*) and p21 (*CDKN1A*), in addition to genes associated with apoptosis (*BAX* and *BCL-2*) and cell migration (*VIM* and *CDH2*) pathways. To do this, RNA was extracted from the transfection cells 72 h upon transfection utilizing the RNX-Plus kit (CinnaGen, Iran). Subsequently, the extracted RNA was reverse transcribed into complementary DNA (cDNA) using the RevertAid First Strand cDNA Synthesis kit (Thermo Fisher Scientific, USA). The primers used in quantitative reverse transcription PCR (RT-qPCR) of the *GUCA2A* gene and the primers used to evaluate the downstream pathways are given in Table 1. For each group, RT-qPCR was conducted in duplicate using SYBR Green and a LightCycler 96 RT-qPCR detection system (Roche, USA) according to the manufacturer’s instructions. Further, changes in gene expression between tumor and adjacent healthy tissues were also evaluated utilizing CRC tissue samples (10 samples) along with their respective adjacent non-cancerous tissues (10 samples) from Iranian patients who visited the Poursina Hakim Research Institute in Esfahan, Iran, during 2021 to 2022. The RNA extraction to RT-qPCR was performed as mentioned above. The study protocol was granted ethical approval by the Ethical Committee of the Hamadan University of Medical Science (ethical code: IR.UMSHA.REC.1399.562).

###  Evaluation of guanylin expression changes following hypoxia treatment 

 After transfection with HRE-p*MUC1*-*GUCA2A* using SPEI-9, the HCT-116 cells were subjected to hypoxic conditions. This was achieved by filling the culture medium up to the top of the well and subsequently sealing it with parafilm. Following a 16 h incubation period post-transfection, alterations in *GUCA2A* gene expression were assessed through RT-qPCR analysis.

###  Annexin V-PI flow cytometry

 To assess apoptosis/necrosis induced by the gene therapy nano-system, the following procedure was followed on two CRC and normal cell lines: a total number of 3 × 10^5^ cells were initially cultured in individual 6-well plates and transfected the following day. After a 72-h incubation period, the cells were harvested using a combination of trypsinization and mechanical scraping (specifically for Vero cells due to their strong cell adhesion), then centrifuged at 1500 g for 5 min. Following this, the cells were subjected to a PBS wash. To the cell pellet dissolved in binding buffer, a mixture containing 10 µL of propidium iodide (PI) dye and 5 µl of Annexin-V dye was added. The samples were then incubated in the dark at room temperature (25 °C) for 10 min. The analysis of the cells was carried out using an Attune NxT Flow Cytometer (Thermo Fisher Scientific, USA) and then FlowJo software. Data were analyzed using FlowJo software. Debris and cell aggregates were excluded based on forward scatter (FSC) and side scatter (SSC) properties, followed by gating on single cells. Apoptosis was quantified using Annexin V-FITC versus PI dot plots, where cells were classified as viable (Annexin V−/PI−), early apoptotic (Annexin V + /PI−), late apoptotic (Annexin V + /PI + ), or necrotic (Annexin V−/PI + ).

###  Cell toxicity experiments

 Cell proliferation assays were conducted to assess the impact of PEI and SPEI-9 nanocarriers with different C/P ratios, along with various gene therapy groups, on both HCT-116 and Vero cells seeded in a 96-well plate with a confluency of 1 × 10^4^ cells. Initially, cells were cultured in 96-well plates until they reached the desired confluence (70-80 %). Subsequently, the cytotoxicity of PEI nanocarriers (at C/P ratios of 0.25 and 1) and SPEI-9 (at different C/P ratios of 0.25, 1, 4, and 8) was evaluated after a 72-h exposure (no removal of medium post-transfection), using the control pEGFP-N1 vector. Additionally, the cytotoxic effects of the gene therapy nano-systems were also investigated following 72 h transfection. To perform this evaluation, 10 μL of MTT solution (5 mg /mL in PBS) was added to each well-containing cell and incubated for an additional 4 h at 37 °C. Following this incubation, the culture medium was carefully removed, and 150 μL of DMSO was added to each well to dissolve the purple formazan crystals. The absorbance was then measured at 490 nm using a microplate reader (Epoch BioTek, USA).

###  In vitro scratch assay

 In this research, we employed the scratch test (wound-healing assay) to evaluate the effect of gene therapy nano-system on the migration and metastatic ability of HCT-116 and Vero cells seeded in a 12-well plate with a confluence of 1 × 10^5^ cells. The procedure involved transfecting cells with various constructs and creating artificial scratches to assess cell viability. Microscopic imaging was conducted at specific time points (0, 24, and 48 h) to monitor the cells’ capability to close the gap created by the scratch. Subsequently, the wound area was quantified using ImageJ software (NIH, USA). Briefly, the wound region was outlined manually, and the wound closure percentage was calculated as:

 Wound closure (%) = [(A₀ – A_t_) / A₀] × 100

 Where A₀ is the wound area at 0 h, and A_t_ is the wound area at each time point.

###  Colony formation assays

 Two different cell lines were seeded into 6-well plates (500 cells/well for the HCT-116 cell line and 1000 cells/well for the Vero cell line). The transfection procedure involving gene constructs was initiated, and the plates were incubated for a minimum of 8 days until visible colonies were formed. Once adequate colony growth was achieved, the plates were subjected to staining with a crystal violet solution to stain the colonies (with at least 50 cells). Subsequently, the number of colonies was quantified and analyzed using ImageJ software.

###  Statistical analysis 

 All data are expressed as the means with SD and the results are representatives of at least three independent experiments. Inferential statistical analyses were performed with an unpaired t-test, Wilcoxon signed-rank test, and one-way analysis of variance (ANOVA) (*P < 0.05; ** P < 0.01; *** P < 0.001; **** P < 0.0001). SPSS 18.0 or GraphPad Prism 9 was used for analysis.

## Results

###  Expression analysis reveals intestine-specific expression of GUCA2A

**Figure 2:**
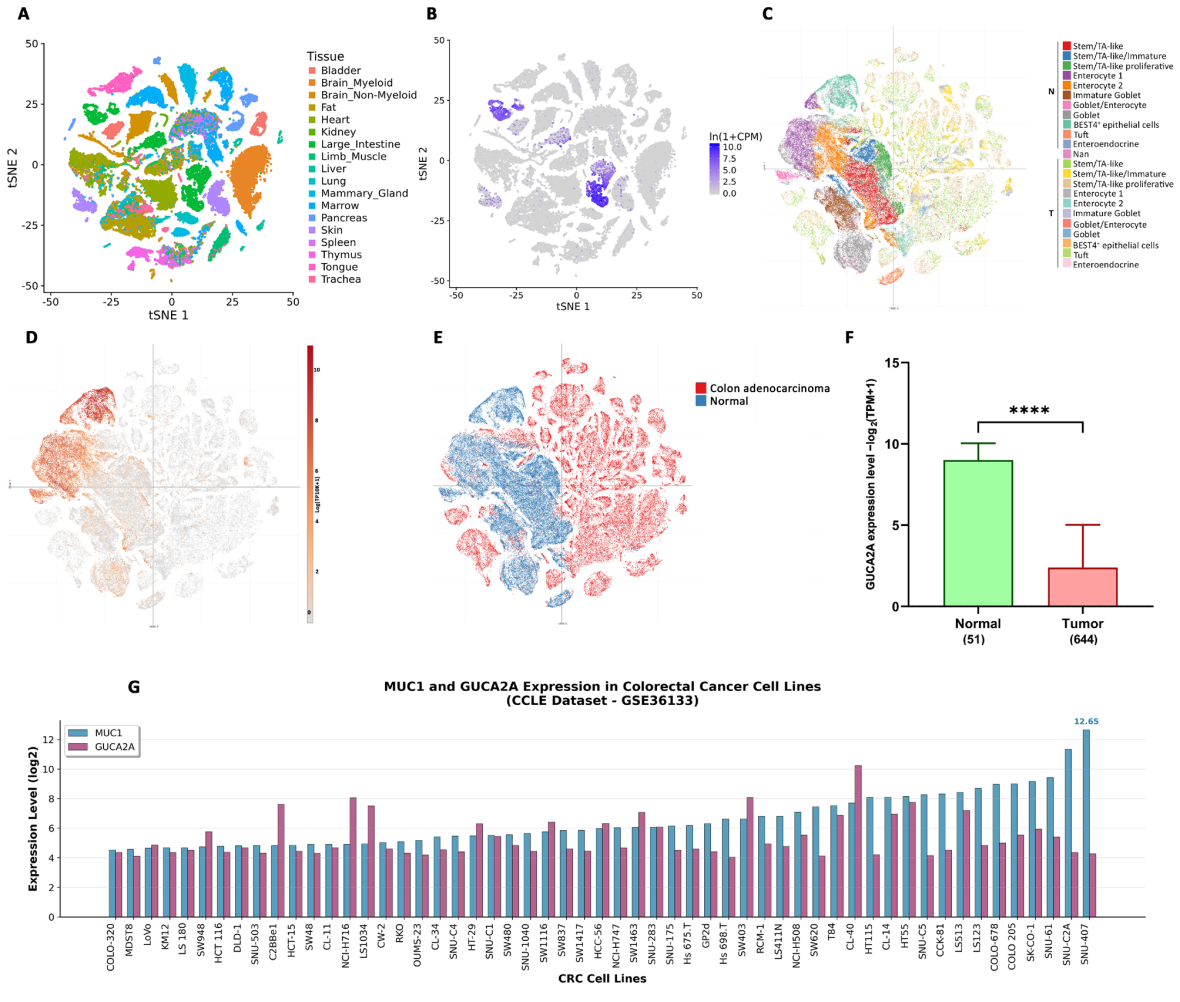
Assessment of GUCA2A expression across multiple datasets. (A–B) Single-cell data show predominant GUCA2A expression in normal large intestine tissues. (C–E) Expression is enriched in enterocytes, BEST4 + epithelial cells, stem/TA-like cells, and goblet cells, but markedly reduced in CRC cells; MUC1 is broadly expressed in goblet and stem/TA-like cells and across CRC cells. (F) GUCA2A levels in normal vs. CRC tissues. (G) Expression of GUCA2A and MUC1 in 55 CRC cell lines

 We investigated the tissue distribution of *GUCA2A* expression utilizing the Tabula Muris and single-cell databases, repositories enriched with valuable single-cell RNA-seq data. The results unveiled an interesting pattern of *GUCA2A* expression primarily within the large intestinal tissue (Figure 2A, 2B). Within the large intestine, GUCA2A showed marked variation across cell types, with the highest expression in enterocytes, BEST4 + epithelial cells, and goblet cells (Figure 2C, 2D), which play pivotal roles in water and ion absorption, nutrient uptake, and vitamin absorption. Conversely, the expression of the *GUCA2A* gene within CRC tumor tissue cells was found to be notably low and, in many cells, entirely lost (Figure 2C, 2F). Moreover, *MUC1* expression showed elevation in goblet cells, immature goblet cells, and intestinal stem cells within normal cell populations as normally mucin is primarily localized to the apical cell membranes in these cell types. In contrast, *MUC1* expression exhibited a more widespread and significant distribution across various tumor cells (Figure 2D, 2C). Additionally, the expression analysis of *GUCA2A* using TCGA COAD-READ datasets displayed significant down-regulation in CRC tissues compared with normal tissues (Figure 2F). Further investigation for the *GUCA2A* and *MUC1* (Figure 2G) was also performed in 55 CRC cell lines, which revealed their distributed expression levels across all CRC cell lines, with a particular emphasis on HCT-116, which aligns with the focus of our study.

###  GUCA2A expression level correlates with poor prognosis in CRC

**Figure 3:**
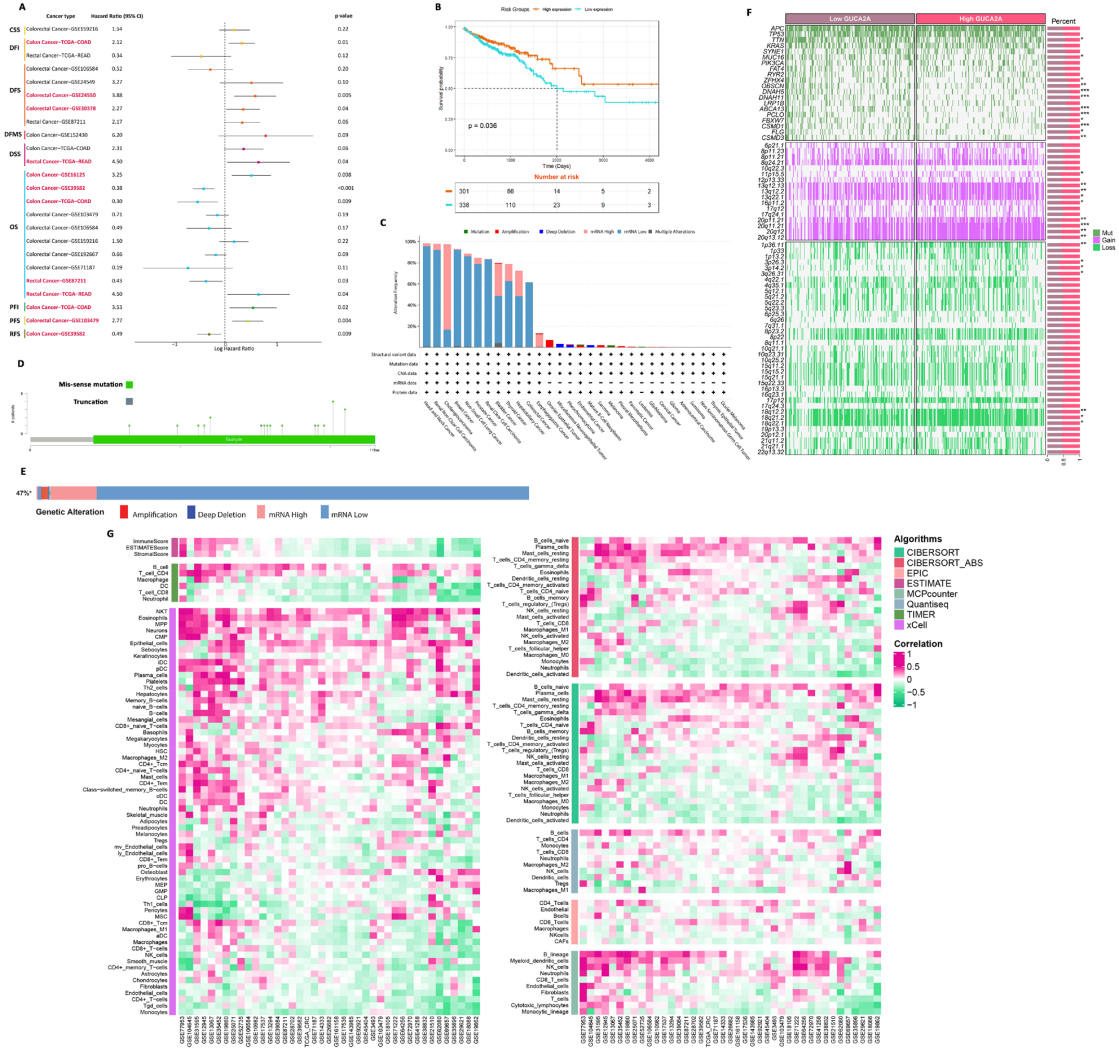
Integrative analysis of GUCA2A. A) Univariate Cox regression across clinical endpoints. B) Kaplan–Meier survival analysis. C–D) Mutational profiles across cancers. E) Hotspot mutations in TCGA. F) Genetic alterations in GUCA2A. G) Correlation between GUCA2A expression and immune cell infiltration

 We employed a univariate Cox regression model to evaluate the association between *GUCA2A* expression and various clinical endpoints in different CRC datasets. Remarkably, reduced *GUCA2A* expression was significantly linked to adverse outcomes across multiple clinical endpoints in CRC (Figure 3A). In this regard, *GUCA2A* expression significantly correlated with worse DFI, DFS, DSS, OS, PFI, PFS, and RFS, which makes it a key gene for the prognosis of CRC. The HR values demonstrate that decreased *GUCA2A* expression is generally associated with a higher risk of unfavorable events such as disease recurrence, progression, and mortality across various clinical endpoints in CRC, except for RFS, where increased *GUCA2A* expression is linked to a reduced risk of disease relapses (Figure 3A). Moreover, the survival curve analysis emphasized that decreased *GUCA2A* expression was also associated with significantly shorter OS time (Figure 3B). Collectively, these discoveries underscore the potential of *GUCA2A* as an innovative and valuable prognostic biomarker in CRC.

###  DNA methylation and alterations of GUCA2A in pan-cancer 

 To investigate the relationship between *GUCA2A* gene mutations and tumor development, we conducted a pan-cancer analysis using the cBioPortal platform, specifically focusing on CRC. The primary alteration type predominantly indicated “mRNA low” in most samples across various cancer types, with a lesser frequency of “mRNA high” alterations (Figure 3D, 3F). Notably, “mRNA low” alterations were observed in over 60% of CRC samples (Figure 3D). Moreover, the somatic mutation frequency analysis of *GUCA2A* revealed missense mutations in several cancer types, including Breast Prostate Adenocarcinoma, Invasive Ductal Carcinoma, Acute Myeloid Leukemia, Hepatocellular Carcinoma, Renal Clear Cell Carcinoma, Uterine Endometrioid Carcinoma, Cutaneous Melanoma, Head and Neck Squamous Cell Carcinoma, and notably in CRC (Figure 3E).

 Copy number variation (CNV) and chromosomal segment alterations were analyzed to identify differences between high and low *GUCA2A* expression groups. CNV data were retrieved and processed using GISTIC 2.0 to identify chromosomal regions with significant gains or losses. Chromosomal segments displaying statistically significant gain-of-function (e.g., 11p15.5, 13q12.13) or loss of function (e.g., 1p36.11, 3p26.3, 3p14.2) were identified by mapping CNV data (Figure3F).

**Figure 4:**
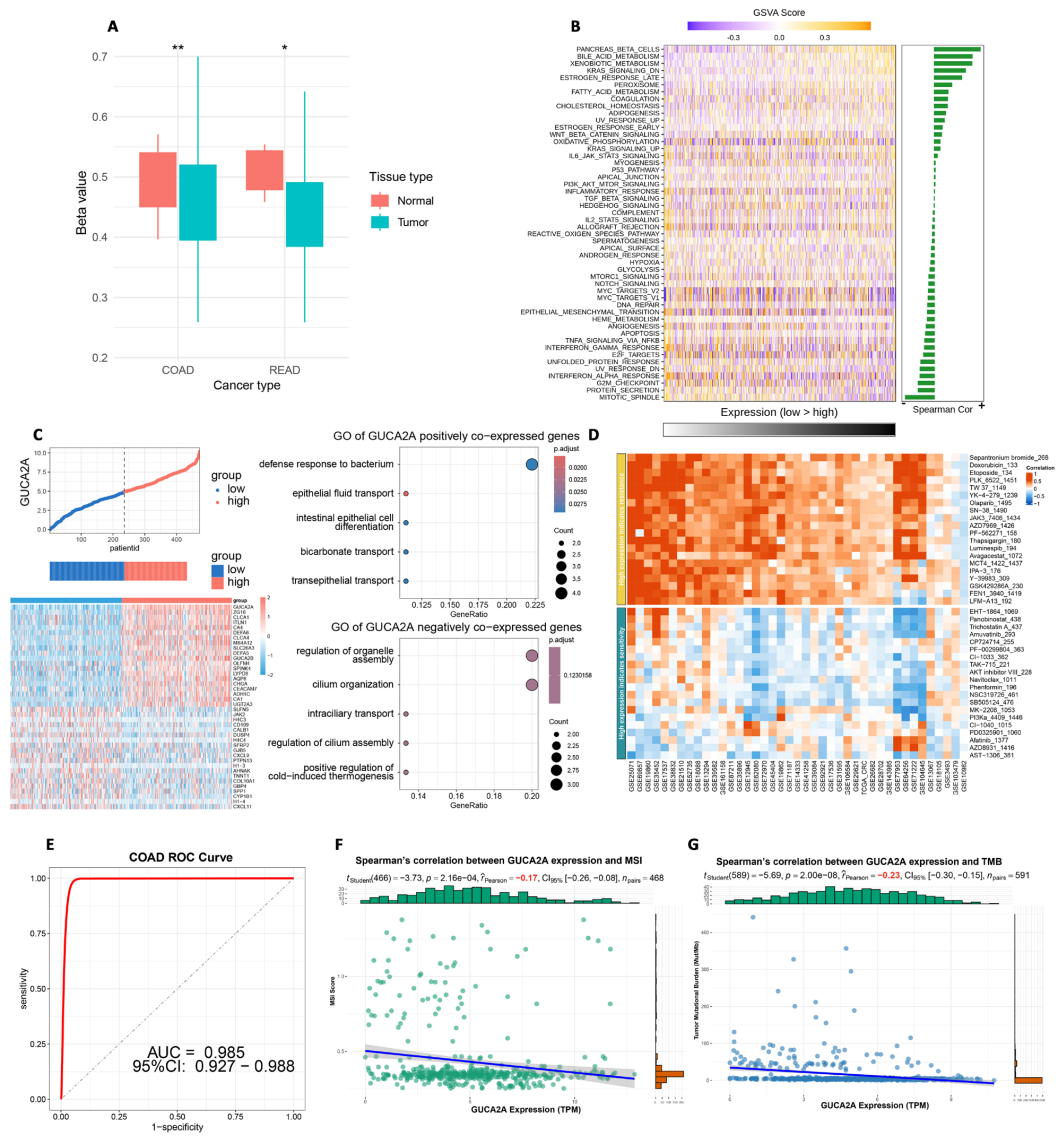
A) Methylation levels of GUCA2A in TCGA COAD-READ. B–C) GSVA and functional correlations in CRC. D) Drug response by GUCA2A expression. E) Diagnostic relevance in CRC. F–G) Association with MSI and TMB

 Furthermore, the analysis of *GUCA2A* gene methylation data unveiled a significant decrease in the promoter methylation level of *GUCA2A* in CRC (Figure 4A). These findings enhance our knowledge of the genetic mechanisms underlying tumor progression and offer further research and potential therapeutic exploration of *GUCA2A*.

###  Immune cell infiltration analysis of GUCA2A in CRC

 To investigate the connection between *GUCA2A* expression and immune cell infiltration, we performed correlation analyses using data from different algorithms. The results showed significant correlations between *GUCA2A* expression and the infiltration levels of macrophages (specifically M1 and M2), T cells (CD4 + , CD8 + , and regulatory T cells), and dendritic cells (Figure 3G). Computational algorithms like ESTIMATE, TIMER, and CIBERSORT highlighted distinct immune profiles, with *GUCA2A* expression strongly linked to increased macrophage M2 polarization and reduced cytotoxic T-cell infiltration, suggesting its role in modulating the tumor microenvironment. Stromal and immune scores calculated by ESTIMATE further supported *GUCA2A*’s impact on immune and stromal cell components. These findings suggest that *GUCA2A* may influence immune evasion mechanisms in CRC and underscore its potential as a biomarker for immune-based therapeutic strategies (Figure 3G). Nonetheless, further clinical investigations are warranted to explore this finding. Moreover, the immune modulator analysis of *GUCA2A* in CRC reveals its dual role in the tumor microenvironment, with significant correlations to antigen presentation markers (HLA-A, HLA-B, HLA-C), chemokines (CCL19, CCL21), and immune inhibitors (PDCD1, TGFB1). These findings suggest *GUCA2A* influences both adaptive immune activation and immune evasion, highlighting its potential as a therapeutic target (Supplementary file 1, Figure S2).

###  GSVA and functional correlation showed key cancer-related pathways linked to GUCA2A

 The relationship between *GUCA2A* expression levels and GSVA scores in CRC is illustrated in Figure 4B. Our biological enrichment analysis revealed distinctive patterns, exhibiting up-regulation in pathways associated with pancreas beta cells, bile acid metabolism, and KRAS signaling with elevated *GUCA2A* expression. Conversely, as *GUCA2A* expression increased, pathways related to mitotic spindle dynamics, protein secretion processes, G2M checkpoint regulation, and interferon alpha responses were downregulated (Figure 4B). These findings provide insights into how *GUCA2A* expression may influence various pathways in CRC, shedding light on potential mechanisms and functional associations. Additionally, the functional analysis of *GUCA2A* in CRC highlights its involvement in diverse biological processes (Figure 4C). Positively co-expressed genes are enriched in pathways related to epithelial barrier function and homeostasis, in contrast, negatively co-expressed genes are associated with intracellular and structural dynamics. These findings suggest *GUCA2A*’s dual role in CRC progression and its potential as a therapeutic target (Figure 4C).

###  Drug response analysis base on GUCA2A expression showed therapeutic potential

 The heatmap illustrates the relationship between *GUCA2A* expression levels and the resistance or sensitivity of various chemical drugs. High expression of *GUCA2A* is associated with differential sensitivity across a broad range of compounds. Red shading indicates a positive correlation with drug resistance, whereas blue shading signifies a relationship with drug sensitivity. This suggests that *GUCA2A* may influence chemotherapeutic efficacy and could potentially serve as a biomarker for predicting drug response in CRC (Figure 4D).

###  Diagnostic Performance of GUCA2A in CRC was exceptional

 The ROC curve evaluated the diagnostic accuracy of GUCA2A expression in CRC. The analysis reveals an area under the curve (AUC) of 0.985, with a 95% confidence interval (CI) of 0.927–0.988. This high AUC value highlights the robust discriminatory power of *GUCA2A* as a diagnostic biomarker for CRC, achieving excellent sensitivity and specificity (Figure 4E).

###  GUCA2A showed significant correlation with MSI and TMB in CRC

 The plots demonstrate the correlation between *GUCA2A* expression and MSI and TMB across multiple cancer types. A significant positive correlation is observed in COAD for both MSI and TMB, indicating that *GUCA2A* expression is closely linked to genomic instability mechanisms in CRC (Figure 4F, 4G). Notably, additional significant associations are observed in stomach adenocarcinoma (STAD) for both MSI and TMB, as well as in testicular germ cell tumors (TGCT) for MSI (Figure 4F, 4G). These findings suggest that *GUCA2A* may influence the mutational and instability profiles of CRC and other cancers, underscoring its potential role in broader genomic instability-associated pathways.

###  Construction of guanylin-expressing constructs

 Following the amplification and isolation of the CDS region of the *GUCA2A* gene (Supplementary file 1, Figure S1A), the obtained fragment and the plasmid constructs (HRE-p*MUC1* and pCMV) underwent digestion using *BamHI* and *XbaI *enzymes. Subsequently, they were purified from the gel and linked together through a ligation process. These resulting plasmids were then introduced into competent bacteria via transformation. Afterward, bacterial colonies were cultured on plates, and the colonies were verified using colony PCR to amplify a 117 bp product (Supplementary file 1, Figure S1B). A single colony was selected for the extraction of plasmids. Finally, the validation of both the HRE-p*MUC1*-*GUCA2A* and pCMV-*GUCA2A* plasmids was performed through Sanger sequencing (Supplementary file 1, Figure S1C). The schematic illustration of the resulting guanylin-expressing constructs is shown in Figure S1D of Supplementary file 1.

###  Structural confirmation of SPEI-9 nanocarrier 

**Figure 5:**
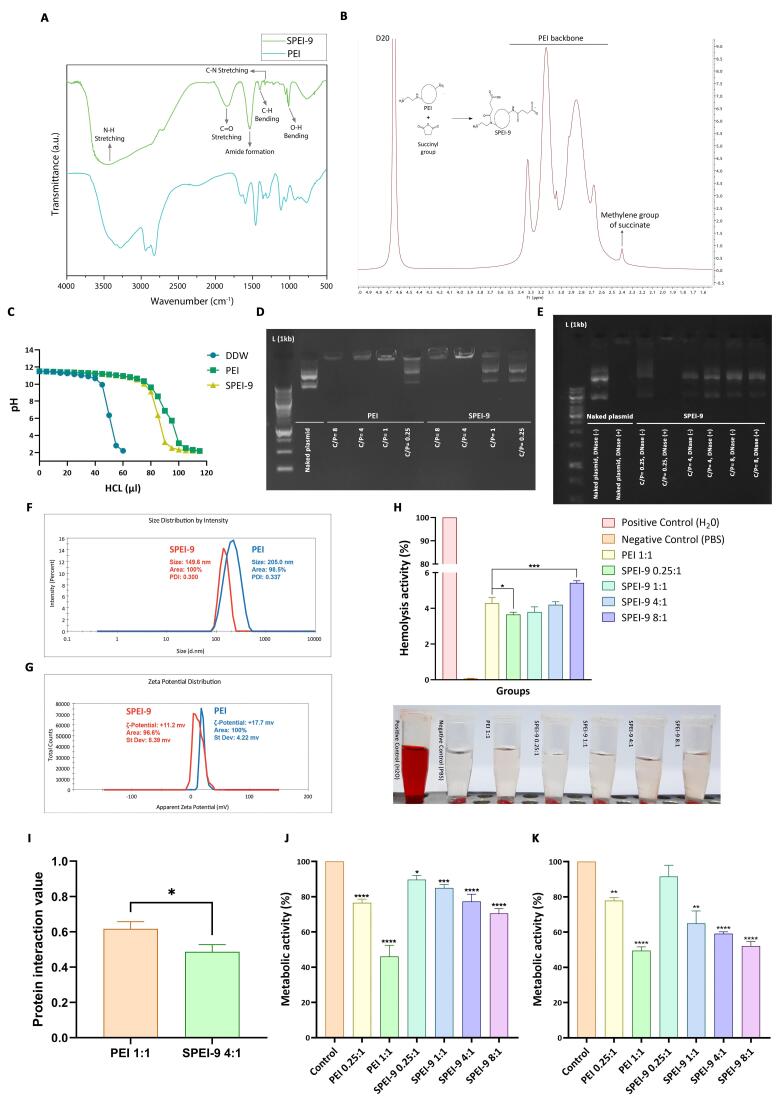
Structural and functional characterization of the SPEI-9 nanocarrier. (A–B) Conjugation validation by FT-IR and NMR. (C–D) Buffering capacity and loading efficiency of SPEI-9 vs. PEI. (E) DNase I protection. (F–G) Particle size and zeta potential. (H) Hemolytic activity. (I) Protein interaction. (J–K) Cytotoxicity in CRC HCT-116 and normal Vero cells

 The FT-IR analysis conducted on basic PEI and SPEI-9 revealed distinct peaks corresponding to the functional groups present in the polymer. These peaks were cross-referenced with the FT-IR spectra library for validation. Notably, the spectrum exhibited a prominent peak at approximately 1760 cm^-1^, which is associated with the stretching vibration of the carbonyl group (C = O) found in succinyl (Figure 5A). This peak serves as an indicator of the binding of the succinyl group to the PEI structure. NMR spectroscopy was employed to perform a structural analysis of SPEI-9. Through this analysis, distinctive peaks associated with various proton environments within the polymer were identified. Notably, the observation of peaks in chemical shifts ranging from 2.5 to 3.5 ppm signified the presence of the PEI backbone (Figure 5B). Additionally, the emergence of new peaks with chemical shifts in the range of 2.3 to 2.5 ppm provided confirmation of the succinyl group’s presence. Consequently, the chemical environment of carbon atoms in the SPEI-9 sample confirmed the successful modification of the polymer.

###  Measuring the buffering capacity of SPEI-9 nanocarrier 

 The buffer capacity of PEI and SPEI-9 was also evaluated in this study. In this regard, compared to the negative control (deionized water), both SPEI-9 and PEI showed significant buffer capacity (Figure 5C). The pH of the solutions remained relatively stable even with the addition of high amounts of acid, indicating their resilience to pH changes. However, since the degree of 9% succinylation was used, SPEI-9 showed relatively lower buffering capacity compared to PEI at concentrations above 80 μL of HCl (Figure 5C). Therefore, the modification of PEI with 9% succinic anhydride largely preserved the suitable buffering properties of the basic nanocarrier, which can be helpful for various applications such as drug delivery and gene therapy, where maintaining a specific pH range is very important.

###  Measuring gel retardation by SPEI-9 nanocarrier

 The DNA loading capacity of both PEI and SPEI-9 was assessed through a gel retardation assay, utilizing various C/P ratios ranging from 0.25 to 8. The results of the gel retardation assay revealed that at lower C/P ratios, specifically 0.25 and 1, SPEI-9 polyplexes exhibited limited DNA loading, as evidenced by their increased mobility during gel electrophoresis (Supplementary file 1, Figure S3C). In contrast, at C/P ratios of 4 and 8, SPEI-9 nanocarriers demonstrated complete plasmid encapsulation, signifying an optimal loading capacity conducive to efficient plasmid delivery (Figure 5D). Conversely, the PEI-based nanocarrier displayed full DNA loading at three distinct C/P ratios: 1, 4, and 8 (Figure 5D). Consequently, the findings from agarose gel electrophoresis underscored the suitability of SPEI-9 nanocarriers at a C/P ratio of 4 and PEI at a C/P ratio of 1 as optimal ratio for gene delivery applications.

###  DNase protection analysis of SPEI-9

 The experiment involved treating SPEI-9 polyplexes at C/P ratios of 0.25, 4, and 8, along with a control gene construct group, with and without DNase. This simulated the presence of nucleases that could potentially break down genetic material. The results revealed that polyplexes formed at a C/P ratio of 0.25, as well as the plasmid structure, were significantly degraded after DNase treatment, as evidenced by the absence of plasmid bands in agarose gel electrophoresis (Figure 5E) (Supplementary file 1, Figure S3D). This indicated the vulnerability of genetic material when complexed with SPEI-9 at this specific C/P ratio. In contrast, polyplexes formed with SPEI-9 at C/P ratios of 4 and 8 displayed robust resistance against DNase degradation (Figure 5E). The bands corresponding to the gene constructs remained well-defined and intact after DNase treatment, underscoring the effective protection provided by SPEI-9 against enzymatic digestion.

###  Measuring the size and surface charge of SPEI-9 nanocarrier 

 The size and surface charge (zeta potential) of PEI and SPEI-9 nanocarriers were determined to evaluate their physicochemical properties, affecting their stability and interaction with genetic materials. In this regard, the results showed that the polyplexes formed with SPEI-9 had an average size of 149.6 nm with an optimum PDI, which indicates that the polyplexes are relatively homogeneous in size and have good stability (Figure 5F). In contrast, polyplexes formed with PEI had a larger average size of 205 nm, indicating a broader size distribution compared to SPEI-9 polyplexes (Figure 5F). Zeta potential measurements also showed that SPEI-9 polyplexes have a positive surface charge, with an average surface charge of + 11.2 mV (Figure 5G). This positive charge is attributed to succinyl and amine groups in the PEI column, which can interact with the negatively charged genetic material. Positive zeta potential indicates good electrostatic stability and effective complexation potential with nucleic acids. In contrast, unmodified PEI polyplexes showed positive zeta potential with an average value of + 17.7 mV (Figure 5G). This higher positive charge is due to the lack of coverage of amine groups by succinyl. With this positive zeta potential, PEI polyplexes showed good stability and the ability to form complexes with genetic materials.

###  Measuring the effect of SPEI-9 nanocarrier on hemolysis rate 

 The hemolytic activity of PEI and SPEI-9 polyplexes was evaluated to assess their potential cytotoxic effects on RBCs. The hemolysis assay included the incubation of polyplexes with RBCs and measuring hemoglobin release, which acts as an indicator of cell membrane damage and hemolysis. The results showed that the rate of hemolysis increases with an increasing C/P ratio for SPEI-9 polyplexes (Figure 5H). At the C/P ratio of 0.25, the amount of hemolysis was relatively low. However, with the increase of C/P ratio to 1, 4, and 8, the degree of hemolysis also increased gradually (Figure 5H). This shows that higher concentrations of SPEI-9 polyplexes may have more potential to induce hemolysis. In comparison, PEI polyplexes at a C/P ratio of 1 had slightly similar hemolysis rare to SPEI-9 polyplexes at a C/P ratio of 4. This suggests that PEI polyplexes may also have some hemolytic activity. Although to a lesser extent compared to the SPEI-9 polyplex, the C/P ratio was higher than 8 (Figure 5H). These results highlight the importance of carefully selecting the C/P ratio and optimizing the formulation of polyplexes to minimize potential cytotoxic effects, especially in the context of functional *in vivo* gene delivery purposes.

###  Interaction assay of SPEI-9 nanocarrier with BSA protein 

 The interaction of PEI and SPEI-9 polyplexes with BSA was evaluated to assess their protein interaction capabilities. The BSA interaction test included the incubation of polyplexes with BSA and measuring the removal or retention of protein in the supernatant of the interaction reaction using spectrometry. In this regard, the results showed that the SPEI-9 polyplex with a C/P ratio of 4, which was selected based on previous tests for downstream studies, had a lower interaction (with an average of 0.48) with BSA compared to PEI polyplexes of C/P ratio 1 with an average of 0.61 (*P* = 0.017) (Figure 5I). The reduction of protein interaction observed with SPEI-9 polyplex indicates that modification of succinylation of PEI may change its surface characteristics and reduce its ability to interact with serum proteins such as BSA. This can be useful in gene delivery applications, as reduced protein interactions can improve stability in circulation to help transfer with higher efficiency.

###  Cytotoxicity assay of SPEI-9 nanocarrier 

 Cytotoxicity assessment of PEI and SPEI-9 was performed in two cell lines, HCT-116 and Vero. In this regard, PEI in C/P ratio 1 showed the highest cytotoxicity in both cell lines, which indicates its destructive effect on cell viability (*P* = 0 < 0.0001) (Figure 5J, 5K). This can be attributed to the high cationic charge density of PEI, which leads to electrostatic interactions with negatively charged cell membranes. These interactions can eventually cause irreparable damage to the cell membrane and lead to cell lysis or necrosis. In contrast, the cytotoxicity of SPEI-9 in both cell lines at different C/P ratios was relatively lower compared to PEI 1 (*P* = 0.0102, 0.0004, 0 < 0.0001) (Figure 5J, 5K). Reducing the charge density of succinylated polymers may help reduce the deleterious effects on cell viability. HCT-116 cells had the lowest toxicity in the presence of SPEI-9 at all C/P ratios, which indicates higher resistance compared to Vero cells. Furthermore, the cytotoxicity of SPEI-9 increased with increasing C/P ratio, indicating a concentration-dependent effect. Even at the highest C/P ratio (8:1), the cytotoxicity of SPEI-9 was almost equal compared to PEI at a C/P ratio of 1:1 (Figure 5J). Cytotoxicity was observed in Vero cells treated with PEI and SPEI-9 more severely. In this context, it was observed that the cytotoxicity of Vero cells was notably lower when treated with SPEI-9 at a C/P ratio of 0.25:1 in comparison to other ratios. However, as the C/P ratio increased, there was a significant elevation in cytotoxicity among Vero cells (*P* = 0.017, *P* < 0.0001) (Figure 5K). These findings underscore the significance of polymer modification, such as succinylation, in mitigating the cytotoxic effects associated with PEI. The reduced cytotoxicity observed with SPEI-9 implies its potential as a safer alternative for gene transfer applications, particularly in the context of cancer cells like HCT-116.

###  Transfection efficiency assay by SPEI-9 nanocarrier 

**Figure 6:**
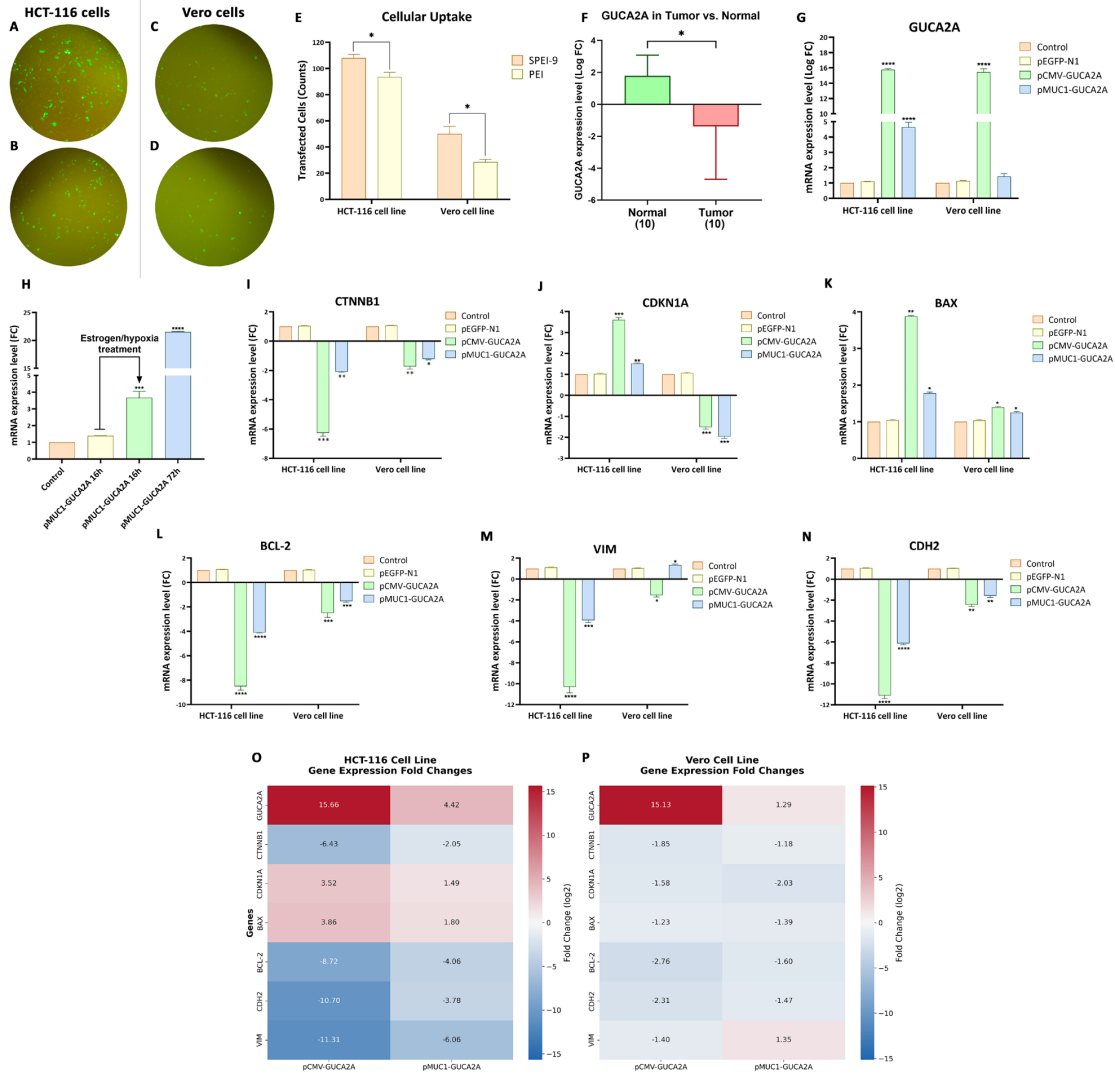
Evaluation of nanocarrier transfection and gene expression. Cellular uptake of the EGFP vector in HCT-116 (A, B) and Vero cells (C, D) using SPEI-9 and PEI, with quantification (E). Differential GUCA2A expression in CRC vs. normal tissues (F) and after guanylin-nanosystem treatment (G) or under hypoxia (H). Expression of CTNNB1, CDKN1A, BAX, BCL-2, VIM, and CDH2 following treatment (I–N). Heatmaps of GUCA2A-regulated genes in HCT-116 (O) and Vero cells (P)

 Analysis of the images acquired via fluorescence microscopy revealed that both SPEI-9 (C/P 4) and PEI (C/P 1) nanocarriers exhibited remarkable efficacy in delivering the pEGFP-N1 plasmid construct, which contains the EGFP protein as a transfection marker. The results indicated that the optimal transfection time for plasmid treatment was 72 h, a widely accepted standard in plasmid transfection protocols. Notably, the transfection rate achieved by the SPEI-9 nanocarrier surpassed that of PEI in both the cancer cell lines HCT-116 (Figure 6A, 6B, 6E, *P* = 0.0229) and the normal Vero cell line (Figure 6C, 6D, 6E, *P* = 0.0373). Consequently, the SPEI-9 nanocarrier, administered for 72 h, was selected for subsequent assessments in cell culture studies.

###  Cell culture study design 

 After conducting different studies involving PEI and SPEI-9 nanocarriers at varying C/P ratios, we have determined that SPEI-9, due to its significantly lower toxicity and superior transfection efficiency during the 72-h treatment, is the optimal choice for subsequent cell culture studies. Furthermore, considering its effective loading at a C/P ratio of 4, along with its reduced toxicity compared to a C/P ratio of 8, we have selected this C/P ratio for the treatment groups in conjunction with various constructs, including pCMV-*GUCA2A* loaded SPEI-9, HRE-p*MUC1*-*GUCA2A* loaded SPEI-9, pEGFP-N1 loaded SPEI-9, and a control group.

###  Evaluation of gene expression changes

 At first, the *GUCA2A* expression level in 10 CRC tissues and 10 adjacent healthy tissues was investigated using RT-qPCR. The results of this study showed a notable decrease and differential expression of *GUCA2A* in tumor tissue compared to the adjacent healthy tissue among different patients (Figure 6F). Additionally, RT-qPCR for *GUCA2A*, p21, β-catenin, *BAX*, *BCL-2*, Cadherin-2, Vimentin, and *GAPDH* (as reference gene) were performed to evaluate the mRNA expression changes upon treatment with different gene therapeutics. In both cell lines, SPEI-9 loaded with pCMV-*GUCA2A* showed remarkable over-expression of guanylin hormone (≈ 15-fold increase in logFC, *P* = 0 < 0.0001) (Figure 6G). While SPEI-9 nanocarrier loaded with p*MUC1*-*GUCA2A*, shown a lower level of increased expression than pCMV-*GUCA2A* in HCT-116 (≈ 5-fold increase in logFC, *P* = 0 < 0.0001) and much lower level of increased expression in Vero cells (not significant), indicating moderate but specific expression of guanylin in cancer cells lines (Figure 6G). This result is consistent with the tumor-specific nature of the *MUC1* gene promoter, which directs the expression of guanylin specifically in tumor cells and minimizes its expression in normal cells.

 In addition to measuring expression changes by the gene constructs, the effects of inducing gene expression by the HRE cassette were also evaluated. We chose the treatment time with and without the effects of hypoxia for 16 h, which showed a significant increase in this period compared to the untreated group (≈ 3.5-fold increase in fold change, *P* = 0.0004) (Figure 6H).

 In this regard, the pCMV-*GUCA2A* group showed a significant decrease of ≈ 6-fold (*P* = 0.0005) and ≈ 1.5-fold (*P* = 0.0023) decrease in β-catenin mRNA levels in HCT-116 and Vero cells, respectively (Figure 6I). Regarding the p*MUC1*-*GUCA2A* gene construct, it showed a significant decline of ≈ 2-fold (*P* = 0.002) and ≈ 1-fold (*P* = 0.113) in both HCT-116 and Vero cell lines, but with less intensity (Figure 6I). Concerning p21, a significant increase in the expression of the pCMV-*GUCA2A* gene construct (*P* = 0.001) compared to p*MUC1*-*GUCA2A* (*P* = 0.0019) was observed for the HCT-116 cell line (Figure 6J). However, in Vero cell line, both gene constructs showed ≈a 1-fold decrease in p21 mRNA level, that this result can be based on the fact that in this cell line the expression of p21 is naturally reduced (Figure 6K).^29^

 The impact of guanylin overexpression on the expression levels of two key apoptotic regulators—BAX, a pro-apoptotic protein, and BCL-2, an anti-apoptotic protein, was also investigated. In this regard, both gene constructs of pCMV-*GUCA2A* and p*MUC1*-*GUCA2A* showed a significant increase in the expression of the apoptosis-promoting gene, BAX, for the HCT-116 cell line (*P* = 0.0040 and *P* = 0.0173) (Figure 6K). These changes in the pCMV-*GUCA2A* group were more intense (≈ 2-fold), which is caused by the significant difference in guanylin expression. On the other hand, in the Vero cell line, this difference in expression was observed with less severity for both pCMV-*GUCA2A* (*P* = 0.0199) and p*MUC1*-*GUCA2A* (*P* = 0.0493) treatment groups (Figure 6K). Moreover, BCL-2 levels have shown a significant decrease in both gene constructs in HCT-116 (*P* = 0 < 0.0001) and Vero (*P* = 0 < 0.001) cell lines (Figure 6L). However, these changes were more intense for pCMV-*GUCA2A* compared to p*MUC1*-*GUCA2A*, with tumor-specific promoters (Figure 6L).

 The results from above were also similar to the expression of Vimentin and N-cadherin, two key genes involved in epithelial–mesenchymal transition (EMT) pathway, due to the strong but non-specific CMV promoter and the moderate but tumor-specific promoter *MUC1*. Regarding Vimentin, the results showed a significant decrease in expression in the HCT-116 cell line for both treatments (*P* = 0 < 0.0001) (Figure 6M). For the Vero cell line, the pCMV-*GUCA2A* group showed a lower expression decrease (*P* = 0.0156) (Figure 6M). On the other hand, in the case of p*MUC1*-*GUCA2A* treatment, this gene showed a tiny increase (*P* = 0.0142). Additionally, for N-cadherin in both HCT-116 and Vero cell lines, both treatment groups showed a significant decrease in expression (*P* = 0 < 0.001), while for the Vero cell line, the changes in the mRNA level were less intense than in the HCT-116 cancer cell line (Figure 6N). Finally, heat maps were generated to visualize the expression patterns of GUCA2A and its six downstream genes in both cancerous and normal cell lines, highlighting the differences in expression distribution between the two (Figure 6O, 6P).

###  Evaluation of apoptosis induction upon guanylin-expressing nano-system

**Figure 7:**
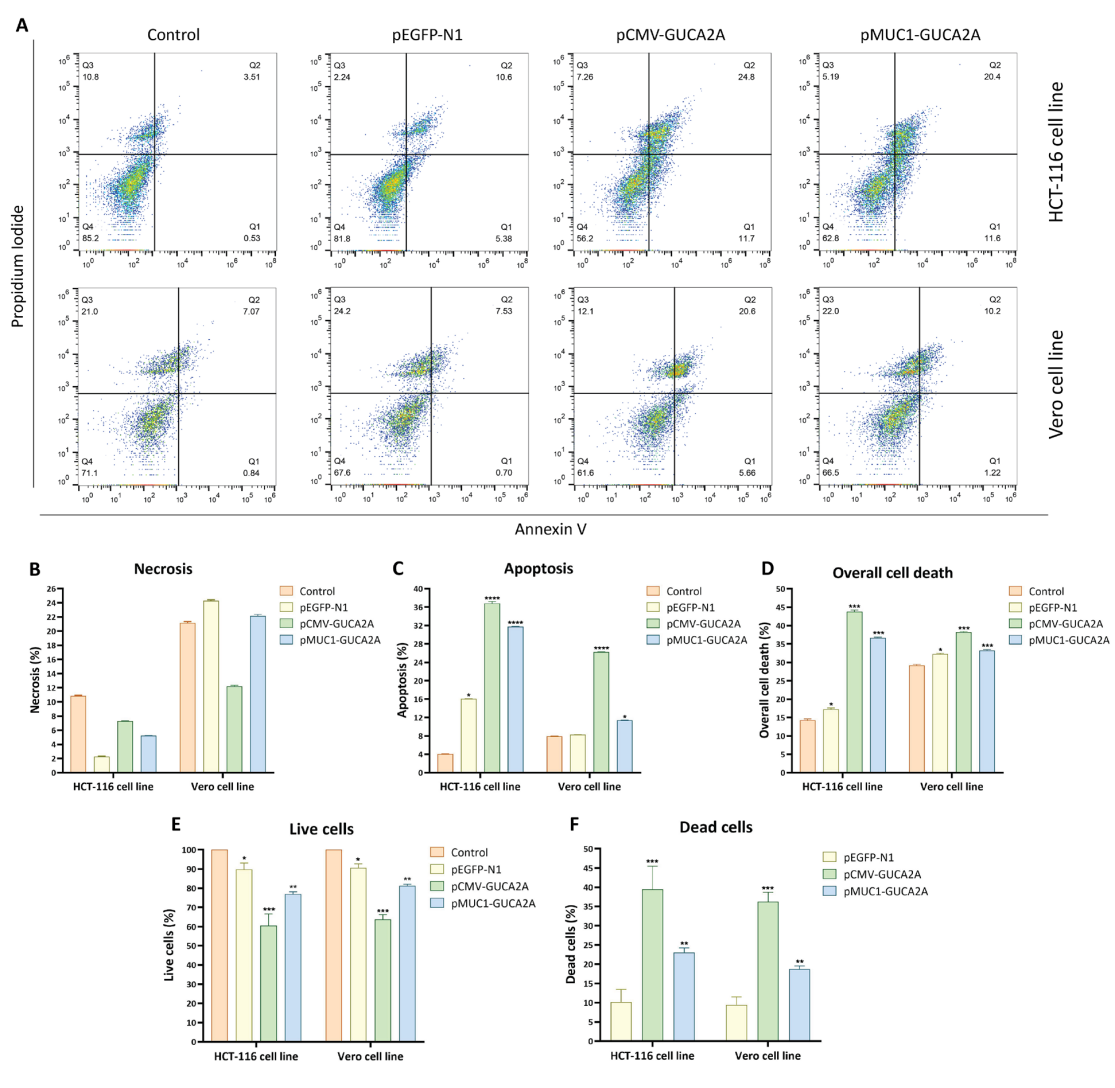
In vitro analysis of apoptosis and cytotoxicity induced by guanylin-expressing vectors. (A) Annexin V/PI staining in HCT-116 and Vero cells. (B–D) Quantification of apoptosis, necrosis, and overall cell death. (E–F) Live and dead cell counts following treatment with pCMV-GUCA2A and HRE-pMUC1-GUCA2A vectors

 To assess the anti-tumor effects of the gene therapeutics, we measured the percentage of induced apoptosis using the Annexin-PI kit and analyzed the results in three categories: necrosis, apoptosis, and total cell death. In this context, we observed minimal necrosis in HCT-116 cancer cells because of the genetic constructs (Figure 7A, 7B). However, in normal Vero cells, necrosis reached up to 25% (Figure 7A, 7B). This relatively higher necrosis percentage in Vero cells can be attributed to their strong adhesion to the culture plate. Additionally, mechanical scraping with a cell scraper contributed to the induction of necrosis. Furthermore, both gene constructs exhibited significant induction of apoptosis in HCT-116 cancer cells (*P* = 0.0001), with approximately 35% for pCMV-*GUCA2A* and approximately 30% for p*MUC1*-*GUCA2A* (Figure 7A, 7C). In Vero cells, the induction of apoptosis was relatively lower, approximately 25% for pCMV-*GUCA2A* (*P* = 0.0001), and even less in the p*MUC1*-*GUCA2A* group, approximately 11% (*P* = 0.0120) (Figure 7A, 7C). Finally, the overall assessment of cell death, which included both early and late apoptosis and necrosis, revealed similar patterns to apoptosis and necrosis (Figure 7D).

###  Evaluation of cytotoxicity upon guanylin gain-of-function 

 Following the evaluation of guanylin expression through RT-qPCR, we assess the impact of different gene constructs delivered via SPEI-9 on cell viability and potential cytotoxicity. In the case of HCT-116 cells, it was evident that cell viability significantly decreased in both the pCMV-*GUCA2A* (*P* = 0.0001) and p*MUC1*-*GUCA2A* (*P* = 0.0030) groups when compared to the control and SPEI-9 groups (Figure 7E). Conversely, the cytotoxicity induced by the gene therapeutics markedly increased in both groups when applied to HCT-116 cell lines. In normal Vero cells, the cytotoxicity induced by pCMV-*GUCA2A* was higher in comparison to p*MUC1*-*GUCA2A* (*P* = 0.0001) (Figure 7F). The subsequent increase in cytotoxicity observed with p*MUC1*-*GUCA2A* was relatively lower than that associated with pCMV-*GUCA2A* (*P* = 0.0019), aligning with expectations (Figure 7F).

###  Evaluation of cell migration ability 

**Figure 8:**
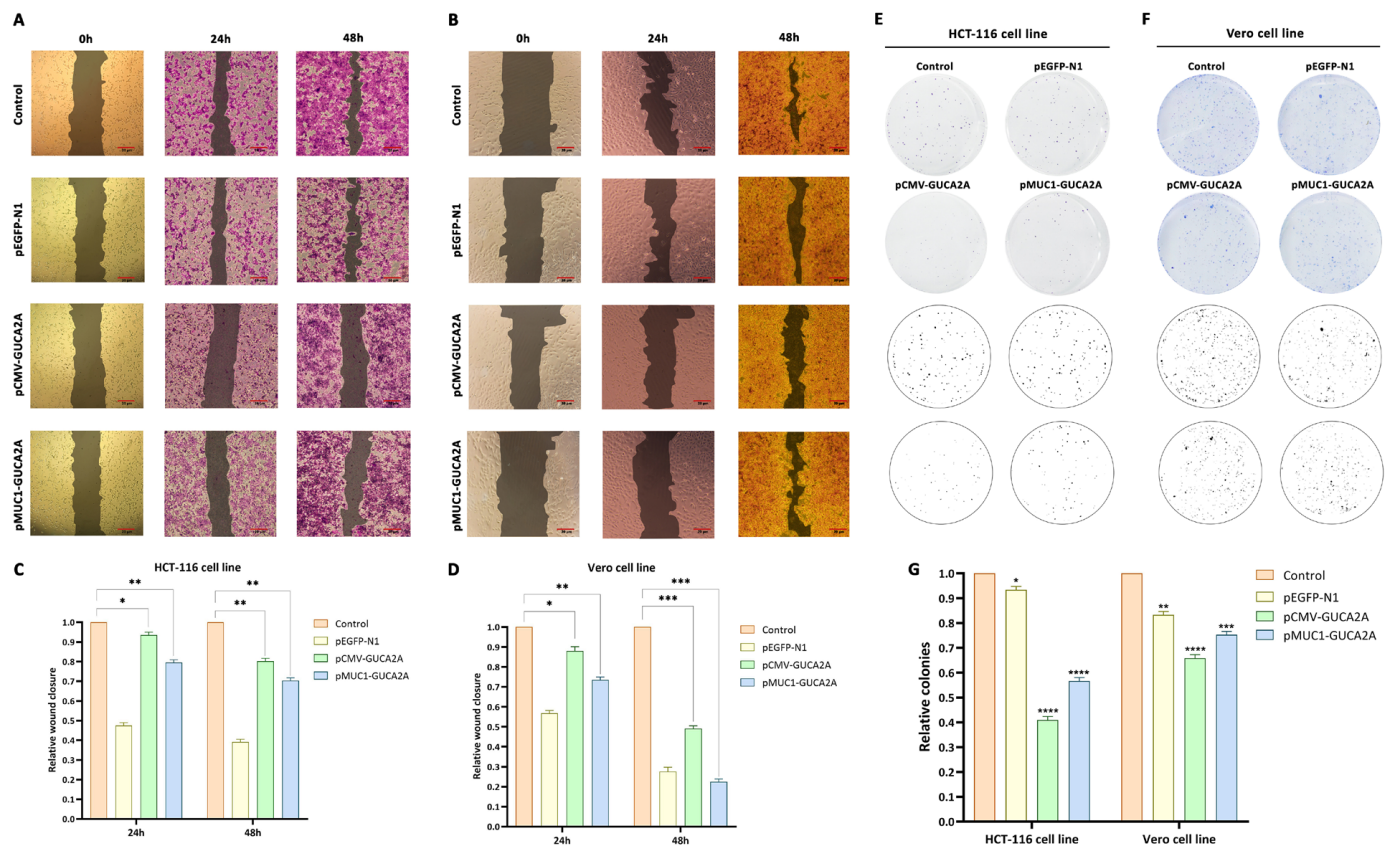
Impact of guanylin-expressing vectors on cellular migration and colony formation. Migration of (A, C) HCT-116 and (B, D) Vero cells; colony formation of (E, G) HCT-116 and (F, G) Vero cells

 To investigate the inhibitory effect of gene constructs on cell migration, a scratch or wound-healing assay was performed. The results of this test showed that pCMV-*GUCA2A* had more substantial anti-migration effects compared to the p*MUC1*-*GUCA2A* vector. In this regard, after 24 and 48 h after treatment in HCT-116 cancer cells, the scratch assay was performed in the group treated with guanylin expression constructs, cell migration at a much lower speed than the nanocarrier group containing gene constructs control and group without treatment were performed (Figure 8A, 8C). Interestingly, these effects in Vero cells were accompanied by a significant decrease in the inhibition of cell migration by treatment with the p*MUC1*-*GUCA2A* gene construct, which results from the low expression of guanylin (Figure 8B, 8D). It is noteworthy to mention that in part A, 24 h and 48 h are separately seeded, treated, and colored, and the control group shown corresponds to the 24 h group.

###  Evaluation of the guanylin-expressing nano-system on colony formation 

 In this test, the effect of different treatments through genetic constructs on the process of colony formation from a seeded cell to a colony of cells (about 50 cells) was evaluated. The results obtained, like the previous results, indicated stronger inhibitory effects of pCMV-*GUCA2A* treatment compared to the structure containing the specific promoter in the HCT-116 cancer cell line. In this regard, both gene constructs in the HCT-116 cell line showed a significant decrease in the number of colonies (*P* = 0 < 0.0001) (Figure 8E, 8G). Also, in the group of normal Vero cells, far less inhibitory effects of the structure containing the *MUC1* promoter (*P* = 0.0001) than CMV (*P* = 0 < 0.0001) were observed, which is consistent with the previous findings (Figure 8F, 8G).

## Discussion

 The application of gene therapy as a potential treatment for cancer has urged the development of various polymeric nanocarriers. The aim was to enhance non-viral vectors as safe and efficient agents for gene transfer. Among these, the PEI nanocarrier, recognized as a benchmark for polymeric vectors, demonstrates notable gene transfer efficiency in serum-free and *in vitro* conditions. Nevertheless, challenges arise under serum-supplemented conditions that mimic the *in vivo* environment. Specifically, PEI/DNA polyplexes tend to aggregate with serum proteins, leading to a reduction in overall transfection efficiency.^30^

 The approaches employed to enhance transfection efficiency and improve the physicochemical characteristics of PEI nanocarriers encompass the conjugation of PEI with diverse polymers, the incorporation of distinct chemical moieties, and the integration of targeting components. For instance, the coupling of polyethylene glycol (PEG) or a stealth polymer, along with more complex chemical groups, establishes a charge protection layer within PEI/DNA polyplexes. This layer serves to mitigate the excess positive charge of the polycation, preventing nonspecific binding to other proteins.^31^ Nevertheless, while these chemical modifications can alleviate polymer toxicity and mitigate interactions with nonspecific proteins, they may concurrently diminish the efficacy of DNA transfer into the cell by reducing its buffering capacity. Hence, the modifications contribute to enhanced gene transfer efficiency, reduced cytotoxicity, improved stability, and tunable properties, which were explored to make modified PEI a promising candidate for advancing gene therapy applications.^30^

 One approach involves attaching anionic components to PEI to reduce the cationic charge density of polyplexes, thereby mitigating cytotoxicity. Using succinic anhydride as a surface modification agent for this polymer can alter its surface characteristics. Following the surface modification of PEI with succinic anhydride, carboxylic groups are introduced to the polymer surface. These carboxylic groups induce various alterations, encompassing changes in contact angle, hydrophobic properties, dispersibility, and the capacity to modify and enhance the electrical charge of the polymer.^24^ The modification degree of Succinylated PEI (SPEI) can be adjusted by varying the quantity of succinic anhydride employed during the modification process, which ranges from 9 to 55% of modified amines. This variability can result in distinct levels of modification, impacting the properties of the resulting SPEI polymer. Notably, SPEI-9, denoting SPEI with a low degree of succinylation (approximately 9% by polymer weight), usually yields lower charge density. Despite a relatively modest reduction in toxicity, it demonstrates higher gene transfer efficiency compared to unmodified PEI. Due to its efficient DNA condensation and protective attributes against degradation, SPEI-9 emerges as an optimal gene delivery vector.^24,30^ In this regard, in a study conducted by Warriner et al., it was demonstrated that modifying the PEI polymer with varying degrees of succinyl groups diminishes the strength of electrostatic interactions between the plasmid and the polymer. Conversely, as the degree of succinylation increases, nonspecific interactions between the polymer and serum proteins decrease, allowing more polymer to be utilized for efficient DNA loading.^30^

 Additionally, the resultant SPEI-9 polyplex exhibited a size of approximately 150 nm, falling within the optimum range for endocytosis without receptor mediation.^32^ Increased succinylation (from 9% to 55%) reduces electrostatic interactions, leading to lower polyplex density and larger nanocarrier size. Moreover, the ζ potential of the polyplexes remained positive, albeit experiencing a slight decrease attributable to succinylation.^30^

 However, the significance of size in polymer design for gene delivery is often underestimated. Studies reveal that PEI-pDNA polyplexes exceeding 100 nm demonstrate enhanced transfection efficiency compared to smaller counterparts.^33-35^ Several explanations have been suggested to rationalize this observation. Firstly, smaller particles indeed exhibit greater solution stability compared to larger ones, which may lead to higher interactions as they sediment onto cell surfaces. Similarly, centrifuging smaller particles onto cells can achieve a similar effect. Another explanation lies in the role of size in the endocytic cycle. For polymers reliant on buffering the endosome and escaping via the proton-sponge phenomenon, larger complexes resulting from higher polymer weight possess increased buffering capacity. This is evident from the limited benefits observed in transfections with lysosomotropic agents for large complexes, while significant efficiency increases are noted for smaller ones. Additionally, vector size can influence the route of internalization.^36^ Clathrin-coated vesicles measure approximately 200 nm in diameter, necessitating adherence to this constraint for particles entering via this route. Larger particles, on the other hand, opt for clathrin-independent pathways, thereby avoiding harsh acidification and trafficking to lysosomes. Cationic polyplexes tend to aggregate with circulatory components like serum proteins and erythrocytes, resulting in clearance or toxicity.^37^ However, smaller and more neutrally charged polyplexes evade this issue by minimizing electrostatic and non-specific binding interactions. Conversely, large polyplexes face reduced cytosolic mobility and rely on active transportation by microtubular and microfibril networks. Thus, achieving an optimal polyplex size entails balancing favorable endocytic trafficking and cellular interactions while optimizing cytotoxicity and cytosolic mobility.^38^

 It is also possible that the end groups of carboxyl succinate may induce a hydration layer, protecting the nanocarrier against serum proteins. However, similar to PEG derivatives, an elevation in the degree of succinylation (45 or 55 degrees) may lead to diminished interactions, stemming either from electrostatic repulsion or physical shielding through hydrated branches. This, in turn, could enhance the polymer’s potential to cause damage to the cell membrane, ultimately associated with a decrease in effective gene transfer.^30,39^ Therefore, considering that SPEI with lower degrees of succinylation offers both lower cytotoxicity and more effective gene transfer, and, in contrast, higher degrees of succinylation lead to increased interactions with serum proteins, the current study opted for the minimum degree of succinylation, 9%, on branched PEI. This choice was made to reduce cytotoxic effects and enhance the efficiency of gene transfer.

 Given that prior investigations on PEI succinylation primarily employed 2 kDa linear PEI, this study stands out by conducting comprehensive structural and functional analyses on the SPEI-9 nanocarrier based on a 25 kDa nanocarrier, yielding novel and promising outcomes. The results obtained from structural confirmation, utilizing FT-IR and H-NMR for the SPEI-9 nanocarrier, align with the findings presented in studies conducted by Zaaeri et al^40^ and Warriner et al.^30^

 Furthermore, concerning the efficient loading of genetic material and the protective capability of the SPEI-9 nanocarrier against degradation by the DNase enzyme, the findings align with the broader outcomes of the study conducted by Nouri et al. Specifically, their study focused on a succinic anhydride group-conjugated nanocarrier (PEI-SUC-PEI) with determined structural and functional characteristics. Nouri et al demonstrated that this nanocarrier exhibited superior buffering resistance compared to PEI-SUC and the base PEI. Interestingly, the loading efficiency and resistance to genetic structure degradation by DNase were nearly identical between PEI-SUC-PEI and PEI-SUC. Notably, the study’s results indicated that the PEI-SUC-PEI nanocarrier, benefiting from two PEI groups, facilitated more effective gene transfer at higher C/P ratios compared to other groups.^34^

 In addition, Zintchenko et al conducted a foundational study in 2008 where the PEI nanocarrier underwent modification with various functional groups, including ethyl acrylate (PEI-EA), acetyl (PEI-AC), succinyl (PEI-SUC), and propionic acid (PEI-PROP). These modifications were applied with varying degrees to assess siRNA transfer efficiency and cytotoxicity in HuH-7 hepatoma cells. The results regarding cell viability demonstrated a proportional increase in cytotoxicity with the escalation of modification degree for all four PEI groups. Notably, the cytotoxicity of PEI-SUC and PEI-PROP nanocarriers was significantly lower than of the others. Furthermore, to evaluate the efficacy of siRNA transfer, polymers from each group were examined at different C/P ratios (ranging from 0.5 to 8). Interestingly, among all the polymers tested, PEI-PROP-18 (C/P ratio 8), PEI-EA-31 (C/P ratios 6 and 8), and PEI-SUC-9 (C/P ratios 4, 6, and 8) exhibited the most potent silencing effects of siRNA. Among these, PEI-SUC-9 demonstrated the highest efficiency, highlighting its remarkable capability for effective gene transfer. Considering the cumulative evidence, the nanocarrier based on succinylated PEI with the lowest modification degree, 9%, emerges as the optimal choice for gene transfer due to its minimal cytotoxicity and maximal gene transfer efficiency.^24^

 Guanylyl cyclase C (GC-C) is a transmembrane receptor prominently expressed apically in intestinal crypts and villus cells.^41^ The GC-C signaling pathway has emerged as a promising therapeutic target for widespread gastrointestinal disorders, including irritable bowel syndrome with constipation, chronic idiopathic constipation, and inflammatory bowel disease.^8,42^ Specifically, GC-C activation is facilitated by intracellular hormonal ligands, uroguanylin and guanylin, predominantly expressed in the small intestine and large intestine, respectively. These hormones activate GC-C, setting off a cascade of downstream signaling pathways. These pathways play a pivotal role in regulating fluid and electrolyte homeostasis, maintaining the integrity of the intestinal epithelium, and influencing tumorigenesis.^43^

 Inactivating mutations in *APC* are linked to 80% of CRC tumors and are also prevalent in other gastrointestinal cancers like gastric cancer.^44^ In this subtype of CRCs, the loss of function in both *APC* alleles is a crucial step in tumor initiation. The inability of APC to regulate the stability of β-catenin protein results in uncontrolled β-catenin nuclear signaling, leading to the activation of oncogenic genes. Although the APC/β-catenin signaling pathway is an appealing target for gastrointestinal cancers, achieving therapeutic effects with drug interventions targeting these molecules proves challenging.^45^

 Remarkably, the connection between the GC-C signaling pathway and CRC was initially revealed through population studies, highlighting an inverse relationship between CRC prevalence and enterotoxigenic *Escherichia coli *(ETEC) infections. ETEC infections involve heat-stable enterotoxins that produce STs, ultimately activating the GC-C signaling pathway and causing diarrhea.^12^ Additionally, the GC-C signaling pathway is implicated in CRC by depleting intracellular ligands, guanylin and uroguanylin. In a study encompassing around 300 tumors and their corresponding adjacent normal tissues, guanylin mRNA exhibited a loss of expression in over 85% of tumors compared to the corresponding normal epithelium.^46^ Notably, recent observations in mice suggest that the loss of guanylin is a direct downstream consequence of mutant APC/β-catenin signaling.^14^ Furthermore, the loss of APC heterozygosity (loss of two alleles) is pivotal for the loss of guanylin hormone expression.^6^ Consequently, these findings highlight that the *GUCY2C* signaling pathway, mediated by guanylin and uroguanylin hormones, may be directly associated with APC/β-catenin mutant signaling in CRC tumorigenesis. Thus, investigating the gain-of-function of these two hormones holds promise for advancing CRC treatment, representing the primary objective of this study.^47^

 Furthermore, based on our prior study involving an integrative transcriptome analysis, we identified the peptide hormone guanylin as the primary therapeutic target for the gain-of-function studies.^5^ Subsequently, guanylin was amplified and cloned into gene constructs containing CMV and *MUC1* promoters. Conversely, considering the synthesis and characterization of the SPEI-9 nanocarrier as an efficient and safe gene delivery agent for gene constructs, diverse cell culture studies were conducted to assess the anti-tumor effects of this therapeutic system.

 To assess the cancer-specific expression and potential off-target effects of our gene therapy construct, we selected Vero cells, derived from African green monkey kidney epithelial cells, as the non-cancerous control. These cells were specifically chosen because they lack endogenous expression of human MUC1, the tumor-specific promoter used to drive GUCA2A expression in our construct. Although direct experimental confirmation of MUC1 absence in Vero cells is limited, prior studies employing MUC1-targeted therapies, particularly in the context of oncolytic virotherapy, have consistently utilized Vero cells as a MUC1-negative baseline model to demonstrate selective activity in MUC1-positive cancer cell lines (e.g., HCT-116).^48^ This selection allowed us to validate that GUCA2A expression was tightly regulated by the MUC1 promoter and did not occur in non-cancerous cells, highlighting the targeting specificity and biosafety of our therapeutic approach. Moreover, Vero cells are widely used for nanoparticle transfection studies due to their robust growth, high transfection compatibility, and predictable morphology, providing a stable and well-characterized model for comparative analysis against CRC cells.^49^

 The selection of cell models in this study was guided by both scientific rationale and logistical limitations. HCT-116 cells were prioritized as they represent an aggressive, undifferentiated CRC model with high Wnt/β-catenin activity, APC mutation, and detectable MUC1 expression, making them highly relevant for assessing guanylin-based therapeutic interventions. For normal controls, Vero cells were used, largely due to accessibility and budget constraints. Although Vero cells are non-colonic and lack MUC1 expression, their very low baseline expression profile allowed us to model non-specific background effects and distinguish tumor-specific responses.^50,51^ These features made HCT-116 and VERO cell lines, suitable models for evaluating the functional impact of the HRE-pMUC1-GUCA2A construct under hypoxic conditions that resemble the real microenvironment. While we recognize the value of including additional CRC cell lines with diverse molecular features, this remains a key objective for future investigations to capture tumor heterogeneity and further validate therapeutic relevance.

 Initially, to validate the transfection efficiency, the SPEI-9 nanocarrier, with a C/P ratio of 4 and loaded with pCMV-*GUCA2A* and p*MUC1*-*GUCA2A* gene constructs, was applied to HCT-116 cancer cells and normal Vero cells. The optimal transfection period of 72 h was chosen to induce maximal peptide hormone expression within the cells. The results of the transfection process, as indicated by *GUCA2A* mRNA expression levels, revealed that the pCMV-*GUCA2A* gene construct exhibited significantly higher expression with lower specificity compared to the construct containing the *MUC1*-specific promoter. It can be inferred that the efficacy of the *MUC1* promoter is contingent on its tissue-specific expression in the relevant cancer. For instance, in a study by Farokhimanesh et al, the PEI nanocarrier loaded with a gene construct containing the *MUC1* promoter and encoding the pro-apoptotic gene truncated *BID* (tBid) demonstrated specific and elevated expression in breast cancer in contrast to the construct with the CMV promoter. Their findings suggested that this heightened and specific expression could induce apoptosis in breast cancer cells (MCF7, T47D, and SKBR3) with minimal impact on normal AGO skin fibroblast cells. Moreover, the induction of expression through the specific *MUC1* promoter in the CRC cell line HT-29 exhibited a notable increase, albeit less evident than in breast cancer cell lines. Considering that the expression of *MUC1* in this cell line differs from HCT-116, it holds more potential for inducing expression.^21^ However, according to diverse investigations, HCT-116 cells are identified as non-differentiated and highly aggressive, with a p53 mutation occurring in the advanced stages of cancer and this cell line exhibited a low expression profile for *MUC1*. In contrast, HT-29 cells are recognized as more differentiated and less aggressive cell lines with mutations in APC observed in the early stages of cancer.^52,53^ Additionally, HT-29 cells can differentiate into enterocytes and *MUC1*-expressing cells. Conversely, given the markedly reduced or absent expression of the guanylin hormone in the progression of CRC and the proven enhanced therapeutic effects in advanced disease stages, the HCT-116 cell line was selected as a tumor model. As a counterpart, the Vero cell line, characterized by very low *MUC1* expression, was chosen as a normal model.^54^

 In addition to assessing the specific induction effects of the *MUC1* promoter, we explored the impact of this stimulus using the HRE upstream of the promoter. Recognizing that prolonged exposure to hypoxia can inhibit apoptosis through therapeutic interventions, a treatment duration of 16 h was chosen based on literature findings. This short period yielded favorable results regarding expression, as evaluated by RT-qPCR. However, it’s important to note that this aspect remained focused on measuring expression levels, with the primary emphasis of the study directed towards investigating the therapeutic effects of the promoters, the *GUCA2A* gene, and the SPEI-9 nanocarrier.^21,55-57^

 The therapeutic rationale for *GUCA2A* overexpression was based on its critical physiological role in maintaining intestinal homeostasis and its marked loss in the early stages of CRC progression, as comprehensively demonstrated in our prior^5^ and present integrative transcriptomic analysis. In our current functional experiments, restoration of *GUCA2A* expression via gene constructs driven by a constitutive CMV promoter and a cancer-selective *MUC1* promoter led to significant modulation of several key signaling pathways associated with tumor suppression. Mechanistically, overexpression of *GUCA2A*, encoding the guanylin peptide hormone, resulted in the reactivation of the GC-C signaling axis. This activation was reflected in suppressing the Wnt/β-catenin signaling pathway, which is known to drive oncogenesis in CRC, particularly in cases with inactivating APC mutations. RT-qPCR analyses showed that β-catenin (CTNNB1) expression was significantly reduced in CRC cells post-transfection, suggesting that *GUCA2A* restoration may re-establish upstream inhibition of β-catenin accumulation and nuclear translocation via cGMP-mediated signaling, consistent with the known physiological function of GC-C. Furthermore, we observed a notable increase in p21 (CDKN1A) gene expression following *GUCA2A* gene therapy. This finding is aligned with previous reports that GC-C pathway activation upregulates p21 and inhibits proliferation in intestinal epithelial cells.^58,59^

 Consistent with prior findings, Blomain et al study showed silencing of *GUCA2A* early in colorectal tumorigenesis by aberrant APC/β-catenin-TCF signaling has been demonstrated in both human tissues and mouse models, confirming the molecular mechanism linking APC loss to *GUCA2A* down-regulation and GUCY2C pathway silencing.^14^ Moreover, in mouse models of CRC, reintroducing guanylin expression restores the activity of the GC-C receptor, leading to increased intracellular levels of cGMP. This reactivation of the cGMP-dependent protein kinase (PKG) signaling cascade has been shown to promote a range of antitumor effects including the inhibition of cell proliferation, induction of cell cycle arrest, and enhancement of apoptosis.^60,61^ Importantly, this signaling pathway negatively regulates the nuclear accumulation of β-catenin, a central effector in the Wnt signaling pathway that drives oncogenic gene expression in CRC.^62^ The reduction of nuclear β-catenin levels by guanylin-mediated cGMP-PKG signaling results in suppression of the transcription of Wnt target genes involved in proliferation and metastasis, thereby attenuating tumor growth.^13,15^

 Additionally, the expression of pro-apoptotic genes such as *BAX* and *caspase-8* was upregulated, while the anti-apoptotic gene *BCL2* was downregulated, culminating in an increased *BAX/BCL2* ratio, a classic indicator of mitochondrial pathway-mediated apoptosis. These results strongly suggest that *GUCA2A* overexpression not only halts cell cycle progression but also triggers programmed cell death in CRC cells. To further assess the effect of *GUCA2A* on cellular invasion and metastatic potential, we evaluated the expression of mesenchymal markers involved in EMT. Notably, Vimentin and N-cadherin expression levels were decreased, implying that *GUCA2A* re-expression may hinder EMT-related processes, thereby limiting tumor cell migration and invasiveness. These findings were particularly prominent in the group transfected with the *MUC1* promoter construct, which allowed for more specific expression in cancer cells and reduced off-target effects in normal cells. Importantly, our results indicate that the observed antitumor effects are directly attributable to *GUCA2A* gene restoration rather than merely to the delivery mechanism. While the nanocarrier played a role in facilitating gene entry into cells, the transcriptional and functional outcomes were a result of *GUCA2A*-driven reactivation of signaling networks that are otherwise dysregulated in CRC. This was further supported by differential expression profiles in both normal and cancerous cell lines, showing selective activation of tumor suppressive pathways in CRC models.

 Further, this restoration has been demonstrated to decrease EMT markers and impair migratory and invasive capabilities of CRC cells *in vitro*, highlighting the pathway’s influence on metastatic potential.^15^ Studies also show that guanylin-induced cGMP-PKG signaling modulates apoptosis regulators such as Bax/Bcl-2 and activates caspases, contributing to enhanced programmed cell death in CRC models.^63^ Moreover, suppressing Wnt signaling has been linked to reversal of EMT, as evidenced by restored expression of epithelial markers like E-cadherin and down-regulation of mesenchymal markers including N-cadherin and Vimentin, leading to reduced invasiveness and metastatic potential.

 The results also demonstrated that elevating guanylin expression, facilitated by both gene constructions, led to suppressed apoptosis induction and diminished expression of genes associated with cell migration pathways. ^64^ In accordance with our results, Chen et al. conducted a study revealing that the long noncoding RNA SRRM2-AS exerts inhibitory effects on angiogenesis in nasopharyngeal carcinoma by activating the MYLK-mediated cGMP-PKG signaling pathway. Their research demonstrated that silencing SRRM2-AS led to increased levels of MYLK, cGMP, PKG, Bax, and Caspase 3, while decreasing levels of VEGF, PCNA, Ki-67, and Bcl-2. Consequently, SRRM2-AS silencing suppressed cell proliferation, colony formation, and angiogenesis, disrupted the cell cycle, and heightened cell apoptosis in nasopharyngeal carcinoma.^65^ Replication of these effects by activation of the cGMP-PKG signaling pathway has also been followed by several other studies in the field.^66-68^

 Notably, in the group treated with the gene construct featuring the specific *MUC1* promoter, there was a quantitative increase in tumor suppressor genes and a decrease in oncogene expression, as observed in the normal Vero cell group. These outcomes signify a specific expression pattern. In this context, Basu et al.’s study yielded interesting findings. In Gucy2c^+/+^ model mice, where the guanylate cyclase C pathway was activated, treatment with bacterial heat-resistant enterotoxin (ST) led to potent antitumor effects. This activation positively regulated the expression of p21 and p38 MAPK genes, culminating in a significant reduction in formed colonies. Notably, these effects were absent in the Gucy2c^-/-^ mouse model, underscoring the critical role of the combined GC-C/cGMP signaling pathway in colorectal carcinogenesis.^59^

 Existing studies in this domain have predominantly centered on activating the GC-C pathway through bacterial ST. For instance, Li and colleagues’ study elucidated the effects of GC-C paracrine pathway activation via oral administration of bacterial ST in a mouse model of radiation-induced gastrointestinal syndrome and different cancer cells. The outcomes highlighted the significant induction of apoptosis in CRC cells (HCT-116) upon GC-C activation by ST. Interestingly, these antitumor effects were contingent on the p53 pathway, as evidenced by their absence in the HCT-116 cell line with an altered phenotype of p53^int-/-^ (with biallelic loss of p53). Moreover, in mouse models of gastrointestinal syndrome, oral administration of bacterial ST substantially reduced in disease symptoms and mortality rates, underscoring its potential therapeutic efficacy.^69^

 While our findings provide valuable insights into the therapeutic potential of guanylin-based gene therapy in CRC, several important limitations must be acknowledged to frame the translational implications of this study. Notably, the current work is confined to *in vitro* experimentation, although significant antitumor effects were observed in HCT-116 cells following transfection with CMV- and *MUC1*-driven *GUCA2A* constructs, the absence of *in vivo* validation limits the generalizability of these results to clinical contexts.

 The observed dual modulation of key signaling pathways, namely the suppression of oncogenic β-catenin and BCL-2 and the up-regulation of tumor suppressors such as p21 and Bax, supports the notion that restoring guanylin expression may regulate multiple mechanisms central to colorectal tumorigenesis. However, the complex *in vivo* tumor microenvironment, characterized by dynamic immune surveillance, stromal interactions, and tumor heterogeneity, is not adequately replicated *In Vitro*. Therefore, the therapeutic impact observed in our model must be interpreted cautiously and considered preliminary until validated in appropriate *in vivo* models.

 The modified SPEI-9 nanocarrier, while demonstrating superior transfection efficiency and lower cytotoxicity compared to conventional PEI carriers *in vitro*, also presents translational challenges. The safety, biodistribution, and immune response to both the nanocarrier and transgene (*GUCA2A*) remain unassessed *in vivo*. The possibility of an immunogenic response, primarily upon systemic delivery of viral or non-self-gene products, could hinder therapeutic efficacy or pose safety risks. Additionally, gene delivery efficiency *in vivo *may be compromised by physiological barriers, including the reticuloendothelial system (RES) clearance, heterogeneous tumor perfusion, and the variability in *MUC1* expression across CRC subtypes.^70^

 From a technical standpoint, the lack of murine *GUCA2A* orthologs with high sequence homology (~68%) limited our ability to test therapeutic constructs in immunocompetent mouse models. Furthermore, logistical constraints, such as the unavailability of facilities for nude or transgenic mouse experimentation, prevented *in vivo* validation in this study. Although theoretical advantages such as tumor accumulation via the enhanced permeability and retention (EPR) effect exist for cationic polymer carriers like SPEI-9, these require empirical verification under physiological conditions.^22^

 While our in vitro findings support the mechanistic plausibility of *GUCA2A* restoration to reactivate the GUCY2C–cGMP–PKG axis and downstream tumor-suppressive programs, a number of translational and ethical hurdles remain to be addressed before clinical application. First, immunogenicity is an important consideration for any gene-therapy platform. Although non-viral polymer systems such as PEI derivatives generally exhibit lower adaptive immunogenicity than viral vectors, PEI and PEI-based formulations can nevertheless trigger innate immune responses, complement activation, or pro-inflammatory cytokine release depending on dose, formulation, and route of administration. These responses may alter nanoparticle biodistribution, reduce repeat dosing potential, or produce local/systemic toxicity. Consequently, thorough immunotoxicity assessments, including cytokine panels, complement activation assays, and repeat-dose studies will be essential in preclinical development.^22^

 Second, off-target expression and promoter specificity require careful evaluation. The *MUC1* promoter confers tumor-associated transcriptional activity in many CRC cells, but low-level *MUC1* expression has been documented in certain normal epithelia and inflamed tissues; thus, promoter leakage remains a realistic safety concern. Strategies to enhance transcriptional confinement, for example, insulator sequences, microRNA target sites that suppress expression in normal cells, or dual-input promoters that require both tumor marker and hypoxia signals should be considered to reduce the risk of ectopic *GUCA2A* expression and unwanted GC-C activation in non-tumor tissues. These approaches will help minimize potential on-target/off-tumor effects of sustained guanylin production.^71,72^

 Third, *in vivo* delivery barriers and biodistribution limit the translation of *in vitro* transfection success to therapeutic benefit. Passive tumor accumulation via the EPR effect is heterogeneous across tumor types and patients and often insufficient to ensure uniform intratumoral distribution. Polyplex size, charge, serum stability, opsonization, and RES clearance all influence circulation time and tumor uptake. Moreover, even when particles reach the tumor interstitium, dense extracellular matrix and elevated interstitial fluid pressure can restrict penetration into tumor cell nests. For these reasons, empirical *in vivo* biodistribution studies, pharmacokinetics, and formulation optimization (e.g., PEGylation, ligand targeting, or stimuli-responsive release) are required to maximize tumor delivery and reduce off-target accumulation.^73,74^

 Fourth, tumor heterogeneity, molecular subtype, and immune contexture will influence therapeutic responsiveness. CRCs are molecularly diverse (MSI vs MSS, varied KRAS/BRAF/APC status, differing tumor mutational burden and immune infiltration), and these variables can alter promoter activity, GC-C pathway wiring, and the cell-intrinsic consequences of cGMP signaling. Our systems biology analyses (expression, methylation, single-cell and clinical endpoint correlations) provided a pan-CRC rationale for targeting *GUCA2A*, but future work must stratify response by molecular subtype, particularly MSI status and TMB, and test the SPEI-9/MUC1-GUCA2A system in models that recapitulate this heterogeneity (patient-derived organoids, PDX, and panels of cell lines). Such stratified preclinical evaluation will help define the patient populations most likely to benefit.^75,76^

 Finally, species homology and model selection pose practical challenges for preclinical validation of *GUCA2A* gene therapy. Human and rodent guanylin sequences and their processing/turnover differ sufficiently that murine models may not fully recapitulate human hormone-receptor dynamics or immunogenicity of the human transgene. This limitation complicates interpretation of in vivo efficacy and safety in conventional mouse models and argues for the use of complementary platforms, humanized mice, orthotopic PDX models, or human colonic organoids when assessing cGMP production, PKG activation, p38 MAPK phosphorylation, and systemic effects of ectopic guanylin expression. Where feasible, comparative sequence and functional assays should guide construct design (e.g., codon optimization, use of human vs species-matched peptide sequences) to minimize cross-species artifacts.^43^

 Future studies are essential to address these limitations. *In vivo* investigations using immunodeficient or humanized CRC xenograft models should be prioritized to assess therapeutic efficacy, biodistribution, and potential off-target effects. Evaluating the immune profile elicited by the nanocarrier and transgene will also ensure safety. To enhance tumor specificity and minimize off-target expression, gene constructs incorporating cancer-selective promoters like *MUC1* should be further optimized, potentially in combination with targeting ligands or stimuli-responsive delivery systems. Moreover, incorporating models that reflect intratumoral heterogeneity—such as patient-derived organoids or xenografts—may offer better insight into therapeutic robustness and translational relevance. Nevertheless, we recognize that inclusion of additional CRC cell lines with varying MUC1 expression levels, differentiation states, and mutational profiles (e.g., KRAS, BRAF, APC) would provide a broader perspective on the applicability of this therapeutic approach. Similarly, testing in human normal colon epithelial cells (e.g., NCM460) would yield a more physiologically relevant control. Future research will prioritize validation across multiple CRC models such as SW480 (MUC1-low), Caco-2 (MUC1-high, enterocyte-like), and HT-29 (differentiated, APC-mutant), alongside NCM460 as a normal comparator. This expansion will help confirm the specificity of the MUC1-driven system, address tumor heterogeneity, and improve translational relevance.^77^

## Conclusion

 In our comprehensive pan-cancer analysis, we uncovered novel insights into the expression patterns of the guanylin hormone across different regions of the colon and rectum. Our results indicate that guanylin may play a critical role in CRC development, participating in complex molecular pathways. Notably, guanylin undergoes frequent mutations and widespread depletion in cancerous tissues, which correlates with reduced patient survival. This dysregulation also aligns with impaired immune system interactions, increased cell proliferation, and inhibition of apoptosis. These findings underscore the central importance of the GC-C signaling pathway in maintaining digestive system homeostasis, spanning both the small and large intestines. Disruption of this pathway—whether through receptor alterations or changes in exogenous ligands such as guanylin and uroguanylin—can have severe consequences for intestinal health. Historically, research has largely focused on pathway activation using chemical stimuli, including bacterial ST and related pharmaceutical agents. In contrast, our study presents a novel approach by assessing the antitumor potential of targeted guanylin induction via the safe and efficient transfection of the pMUC1-GUCA2A construct using SPEI-9 as a potent nanocarrier. Observations from both tissue-specific and systemic expression systems suggest that guanylin-based gene therapy represents a promising avenue for future clinical trials in CRC treatment.

## Competing Interests

 The authors declare that there are no conflicts of interest.

## Data Availability Statement

 All data generated or analyzed during this study are either publicly available or can be obtained from the corresponding author upon reasonable request. Publicly available transcriptomic datasets used for *in silico *analyses were retrieved from The Cancer Genome Atlas (TCGA) and the Gene Expression Omnibus (GEO). Specifically, the TCGA datasets include Colon Adenocarcinoma (TCGA–COAD) and Rectal Adenocarcinoma (TCGA–READ), accessible via the GDC Data Portal (https://gdc.cancer.gov/). GEO datasets used include GSE159216, GSE36133, GSE106584, GSE24549, GSE24550, GSE30378, GSE87211, GSE152430, GSE16125, GSE39582, GSE103479, GSE192667, and GSE71187, available at https://www.ncbi.nlm.nih.gov/geo/. Additional analyses were performed using established web platforms including GTEx (https://commonfund.nih.gov/GTEx/), cBioPortal (https://www.cbioportal.org/), DAVID (https://david.ncifcrf.gov/), TIMER2.0 (http://timer.cistrome.org/), SmartApp (http://www.bioinfo-zs.com/smartapp/), and TISIDB (http://cis.hku.hk/TISIDB/index.php). Experimental raw data, such as qPCR results and cytotoxicity assay outputs, are not publicly archived but are available from the corresponding author upon reasonable request.

## Ethical Approval

 All procedures were performed in accordance with the Declaration of Helsinki and approved by the ethics committee of the Hamadan university of medical sciences (IR.UMSHA.REC.1399.562). Informed consent was obtained from all subjects and or their legal guardians. Patient samples were collected from the Poursina Hakim Research Institute (Esfahan, Iran).

## Supplementary Files


Supplementary file 1 contains Figures S1-3.
