# Supplementary material for: MUC1-Driven Guanylin Gene Delivery via Succinylated PEI-9 Nanocarrier for Colorectal Cancer Treatment: An in Silico and In vitro Study
Source: Adv Pharm Bull. 2025 Oct 15;15(4):844–70. doi: 10.34172/apb.025.45741 (PMC13058149; doi:10.34172/apb.025.45741)
Supplement: Supplementary file 1 — contains Figures S1-3. [file apb-15-844-s001.pdf]

Adv Pharm Bull. 2025;15(4):844-S1
doi: 10.34172/apb.025.45741
https://apb.tbzmed.ac.ir

Supplementary Figures:

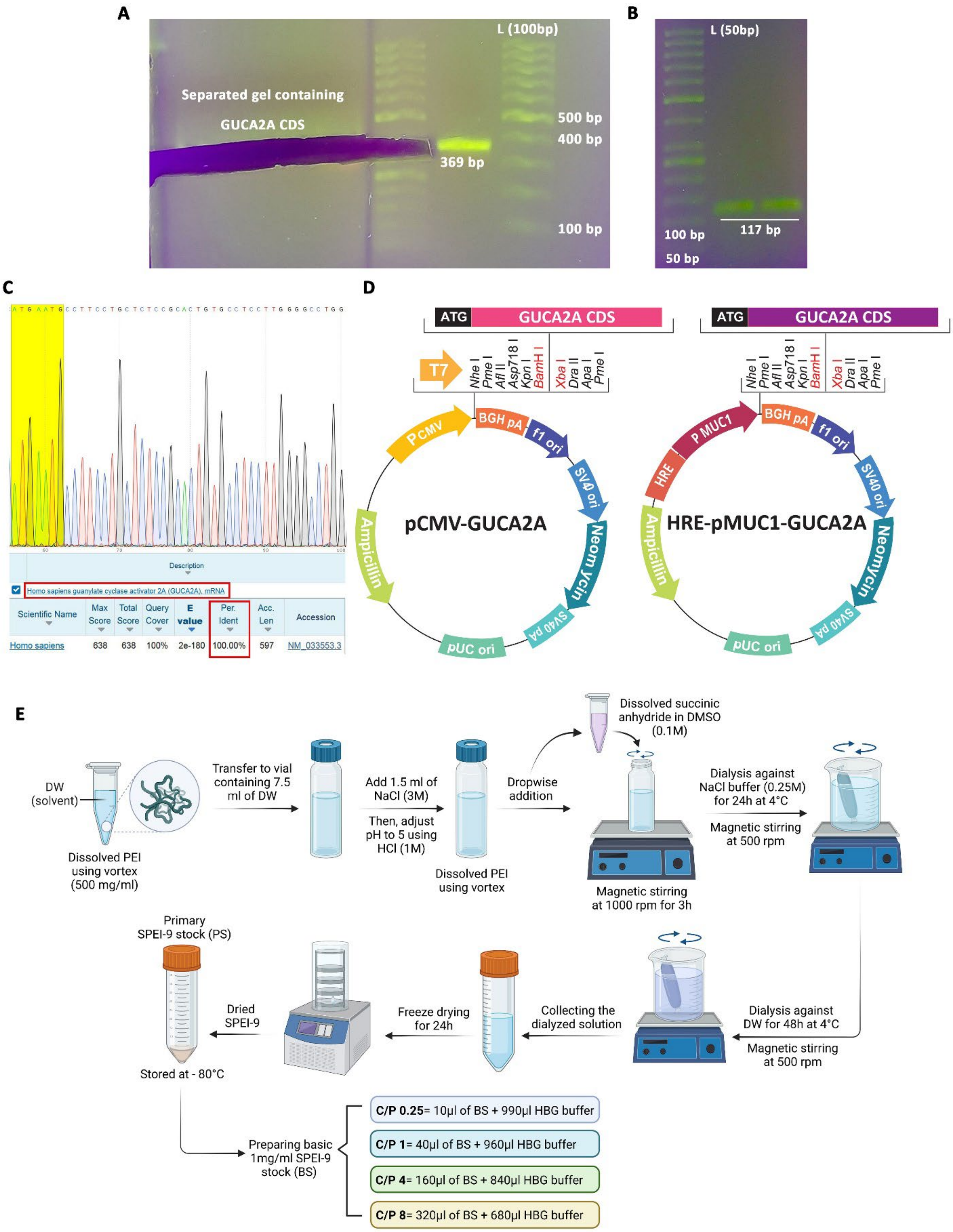

Figure S1. Construction of the guanylin-expressing nano-system. **A)** Amplification and gel extraction of GUCA2A coding sequence (CDS). **B)** Colony PCR validation of the constructed pCMV-GUCA2A and HRE-pMUC1-GUCA2A vectors. **C)** Final validation of the constructed vectors through Sanger sequencing. **D)** Schematic diagram of the constructed pCMV-GUCA2A and HRE-pMUC1-GUCA2A vectors. **E)** Schematic representation of the various steps involved in the synthesis of the SPEI-9 nanocarrier. This schematic is created with BioRender.

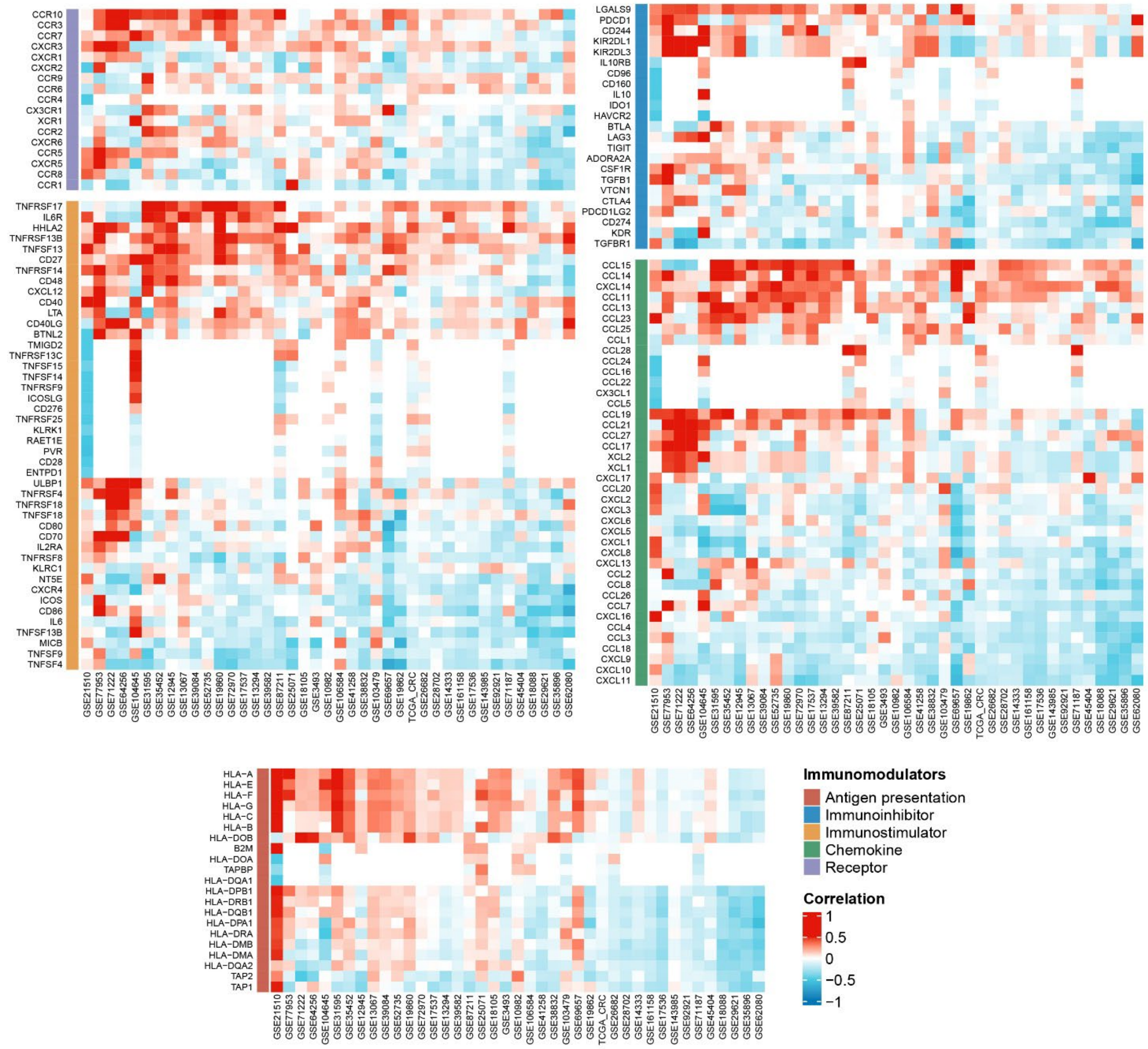

Figure S2. Correlation between GUCA2A expression and different immunomodulators.

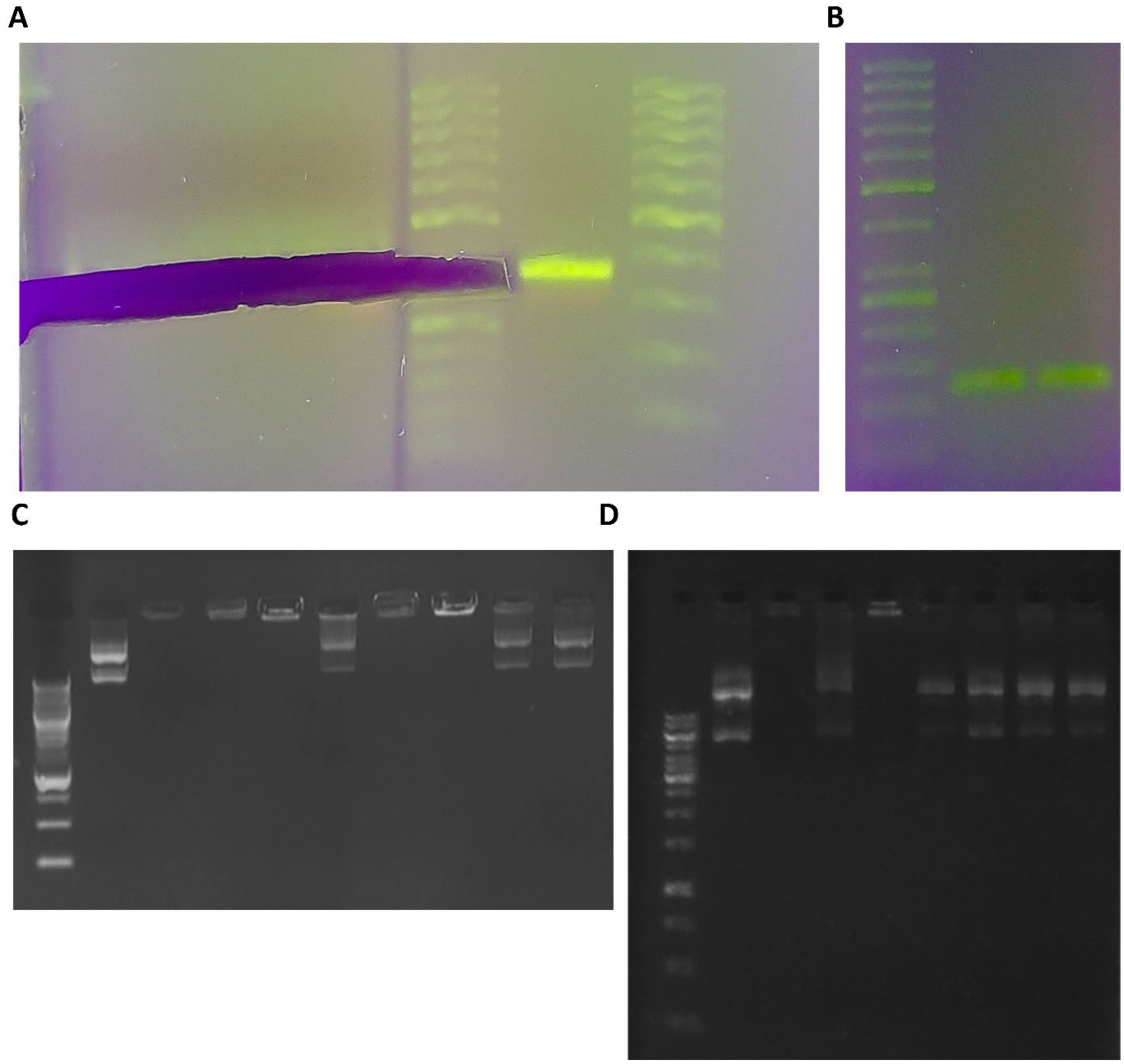

Figure S3. Raw images of gel electrophoresis for: **A)** Amplification and gel extraction of GUCA2A coding sequence (CDS). **B)** Colony PCR validation of the constructed pCMV-GUCA2A and HRE-pMUC1-GUCA2A vectors. **C)** Evaluation of the loading efficiency of synthesized SPEI-9 and PEI. **D)** Assessment of vector protection against degradation by DNase I loaded in SPEI-9.
